# Aerobic methanotrophy increases the net iron reduction in methanogenic lake sediments

**DOI:** 10.3389/fmicb.2023.1206414

**Published:** 2023-07-27

**Authors:** Hanni Vigderovich, Werner Eckert, Marcus Elvert, Almog Gafni, Maxim Rubin-Blum, Oded Bergman, Orit Sivan

**Affiliations:** ^1^Department of Earth and Environmental Sciences, Ben-Gurion University of the Negev, Beer Sheva, Israel; ^2^The Yigal Allon Kinneret Limnological Laboratory, Israel Oceanographic and Limnological Research, Migdal, Israel; ^3^MARUM—Center for Marine Environmental Sciences and Faculty of Geosciences, University of Bremen, Bremen, Germany; ^4^Israel Oceanographic and Limnological Research, National Institute of Oceanography, Haifa, Israel

**Keywords:** aerobic methanotrophy, iron reduction, lake sediments, iron recycling, methylotrophy, methanogenesis

## Abstract

In methane (CH_4_) generating sediments, methane oxidation coupled with iron reduction was suggested to be catalyzed by archaea and bacterial methanotrophs of the order Methylococcales. However, the co-existence of these aerobic and anaerobic microbes, the link between the processes, and the oxygen requirement for the bacterial methanotrophs have remained unclear. Here, we show how stimulation of aerobic methane oxidation at an energetically low experimental environment influences net iron reduction, accompanied by distinct microbial community changes and lipid biomarker patterns. We performed incubation experiments (between 30 and 120 days long) with methane generating lake sediments amended with ^13^C-labeled methane, following the additions of hematite and different oxygen levels in nitrogen headspace, and monitored methane turnover by ^13^C-DIC measurements. Increasing oxygen exposure (up to 1%) promoted aerobic methanotrophy, considerable net iron reduction, and the increase of microbes, such as *Methylomonas*, *Geobacter*, and *Desulfuromonas*, with the latter two being likely candidates for iron recycling. Amendments of ^13^C-labeled methanol as a potential substrate for the methanotrophs under hypoxia instead of methane indicate that this substrate primarily fuels methylotrophic methanogenesis, identified by high methane concentrations, strongly positive δ^13^C_DIC_ values, and archaeal lipid stable isotope data. In contrast, the inhibition of methanogenesis by 2-bromoethanesulfonate (BES) led to increased methanol turnover, as suggested by similar ^13^C enrichment in DIC and high amounts of newly produced bacterial fatty acids, probably derived from heterotrophic bacteria. Our experiments show a complex link between aerobic methanotrophy and iron reduction, which indicates iron recycling as a survival mechanism for microbes under hypoxia.

## Highlights

- Stimulation of aerobic methanotrophy with oxygen levels up to 1% increases the net iron reduction in energy-limited methane-generating lake sediments.
- Iron reduction is performed either by iron-reducing bacteria, such as *Desulfuromonas* or *Geobacter*, or by the methanotrophs themselves in a survival mode.
- Under hypoxia, methanol is not involved as a substrate for the methanotrophs instead of methane.

## 1. Introduction

Methane (CH_4_) is a very efficient and potent greenhouse gas, 28 times more efficient than CO_2_ on a 100-year time scale (Myhre et al., 2013). It is microbially produced in anoxic marine and freshwater settings. Freshwater environments contribute greatly to methane emissions (Bastviken et al., 2011), despite taking up a much smaller portion of the Earth’s surface than oceans (Downing et al., 2006). The methane formed in the sediments can be attenuated by oxidation with available electron acceptors. Methane oxidation is microbially mediated in two fashions, anaerobically or aerobically. Anaerobic oxidation of methane (AOM) in marine sediments is mainly coupled to sulfate reduction via anaerobic methanotrophs (ANMEs; Knittel and Boetius, 2009). In freshwater sediments, which are usually depleted in sulfate, AOM is coupled to other electron acceptors like nitrate, nitrite, metal oxides, and humic substances. It is performed mostly by different ANMEs (mainly by ANME-2) with or without a bacterial partner (Raghoebarsing et al., 2006; Haroon et al., 2013; Nordi and Thamdrup, 2014; Ettwig et al., 2016; Lu et al., 2016; Scheller et al., 2016; Cai et al., 2018; Elul et al., 2021).

Aerobic methane oxidation is found in the oxic-anoxic transition zone (Bender and Conrad, 1994; He et al., 2012), usually at the sediment–water interface or the oxycline in the water column of stratified systems, and is performed by different methanotrophic bacteria (type-I, II, X; McDonald et al., 2008; Trotsenko and Murrell, 2008; Smith and Wrighton, 2019). Generally, during aerobic methanotrophy, bacteria use the enzyme complex methane monooxygenase (MMO) to oxidize methane with oxygen to methanol. The methanol is then oxidized to formaldehyde, which is finally oxidized to CO_2_ (Dalton, 2005).

Interestingly, growing evidence in recent years indicates aerobic methanotrophs and methylotrophs activity below the oxic-anoxic zone in the anoxic hypolimnion of freshwater lakes (Blees et al., 2014; Oswald et al., 2016a) and sediments (Beck et al., 2013; Bar-Or et al., 2015; Martinez-Cruz et al., 2017; Su et al., 2022). Concomitantly, obligate anaerobic microbes, such as methanogens and iron reducers, were observed there (Elul et al., 2021; Van Grinsven et al., 2021; Steinsdóttir et al., 2022; Su et al., 2022).

Three possible scenarios can explain this co-occurrence of aerobic and anaerobic microorganisms; (1) Microlevel oxygen is trapped and survive this environment (Wang et al., 2018). The oxygen is slowly released so it does not poison the obligatory anaerobes. In this case, long-term anoxic conditions will terminate aerobic activity (Vigderovich et al., 2022). (2) Low oxygen levels are continuously produced in the anoxic environment and are immediately used by these methanotrophs. This has been demonstrated for the aerobic bacteria *Methylomirabilis* (NC10), which produce and utilize oxygen during a unique denitrification process to oxidize methane (Ettwig et al., 2010). Similarly, the archaeon *Nitrosopulimus maritimus* is suggested to produce oxygen upon depletion (to 1 nM) to mediate ammonia oxidation (Kraft et al., 2022). Alphaproteobacterial methanotrophs have been shown to survive under hypoxia by utilizing methanobactins to generate oxygen and fuel their methanotrophic activity (Dershwitz et al., 2021). (3) Under hypoxia conditions, aerobic methanotrophs survival is mediated by anaerobic metabolism. Recent experiments with sediments and pure cultures of methanotrophic bacteria show that these methanotrophs can use electron acceptors other than oxygen under hypoxia. The Gammaproteobacterial methanotrophs *Methylomonas* and *Methylosinus* were suggested to perform methane oxidation coupled with the reduction of metal oxides (Zheng et al., 2020). The Alphaproteobacterial methanotroph *Methylocystis* sp., strain SB2 was shown to couple methane oxidation with iron reduction (Dershwitz et al., 2021). *Methylomonas denitrificans* strain FJG1 expresses genes that encode for nitrate reduction (Kits et al., 2015; Orata et al., 2018). In addition, it is suggested that the Gammaproteobacteria *Methylocaldum* can couple methane oxidation to N_2_O reduction in wetland sediments under anoxic conditions (Cheng et al., 2021). It should be noted that the presence of aerobic methanotrophic bacteria in a highly reduced environment without detectable oxygen raises the question whether the environment accounts as anoxic or hypoxic. In this study, we define hypoxia as a reduced environment with down to undetectable oxygen levels (below our detection limit of 1 ppb) but with evidence for active aerobic metabolism. The conditions are anoxic when oxygen is not detected and there is no evidence for aerobic activity.

Lake Kinneret (Sea of Galilee) is a monomictic lake in northern Israel. Its average depth is 24 m, and its maximum depth is 42 m at the center (station A). The lake is stratified between March and December, leading to about 20 m of hypolimnion with undetectable oxygen concentrations most of the stratified period (Adler et al., 2011). Sulfate is depleted in the upper few centimeters of the sediment and the methane zone below is characterized by low redox conditions (−200 mv) and unmeasurable oxygen levels (Eckert and Conrad, 2007). Despite this, *pmoA* gene-bearing methanotrophic bacteria together with *mcr* gene-bearing archaea were suggested to mediate methane oxidation coupled with iron reduction (Bar-or et al., 2017; Elul et al., 2021; Vigderovich et al., 2022) in these sediments. Aerobic type-I Gammaproteobacteria methanotrophs were observed, and evidence for aerobic methanotrophy has been demonstrated by the presence of specific fatty acids, the *pmoA* functional gene, and metagenomic analysis of incubation experiments (Bar-Or et al., 2015, 2017; Elul et al., 2021). These aerobic methanotrophs operate alongside methanogenesis and iron reduction in the methane generating sediments (Elul et al., 2021). This phenomenon was observed also in other sediments of shallow lakes (Martinez-Cruz et al., 2017; Su et al., 2022). Given that *pmoA* activity must involve oxygen (Dalton, 2005), and that it appears only in the natural (fresh) methane-generating sediments and incubations (and not in long-term two-stage incubations; Vigderovich et al., 2022), it seems that remnant microlevels of oxygen would be the most plausible scenario responsible for the methanotrophs’ activity, making this environment hypoxic and not completely anoxic (with remnant oxygen but low enough redox values that enable the life of strictly anaerobes) However, it is unknown how the aerobic microbes survive and whether their survival is linked to the observed iron reduction coupled with methane oxidation in these sediments.

Here, we explored the potential link between aerobic methanotrophy and iron reduction in methane generating sediments by injecting low (micro) levels of oxygen into lake sediment slurries and quantified its effect on net iron reduction. This is by a set of slurry incubations with methane generating sediments of Lake Kinneret amended with ^13^C-labeled methane, hematite, and with and without inhibition of methanogenesis by BES. Finally, we tested whether methanotrophic bacteria can operate under hypoxia by using the potential intermediate methanol as a substrate by another set of slurry incubations with ^13^C-labeled methanol.

## 2. Materials and methods

### 2.1. Study site

The sediments and the extracted porewater used in this study were from the methane-generating depth (below 20 cm from the water–sediment interface from station A). They are mostly carbonatic-clay and contained 7% iron oxides (Vigderovich et al., 2022) and about 3% total organic carbon (TOC). Dissolved sulfate concentrations decrease from about 0.5 mM at the surface sediment to depletion around 10 cm depth, where dissolved Fe(II) appears and increases with depth up to 80 μM at 30 cm depth. Dissolved methane concentrations increase with sediment depth, reaching a maximum of 2 mM at 10–15 cm depth. The concentrations decrease then to 0.5 mM at 30 cm depth. The dissolved organic carbon (DOC) concentrations in the porewater increase with sediment depth, from ~6 mg C L^−1^ at the sediment–water interface to 17 mg C L^−1^ at 25 cm depth (Adler et al., 2011; Sivan et al., 2011; Bar-Or et al., 2015).

### 2.2. Sediment sampling

Sediment cores were collected using a gravity corer on four day-long sampling campaigns (Table 1), on the research vessel *Lillian*, between 2017 and 2021 from station A in the center of the lake (water depth 42 m). In each campaign, 1–2 Perspex cores of 50 cm long were collected for the incubation experiment, and another 10 cores were collected for porewater extractions. For the porewater extraction, sediment from the methane-generating zone (sediment depth > 20 cm) of each core was transferred to a 5 L plastic container onboard. The cores and the container were brought to the lab, and the cores were kept at 4°C, while porewater was extracted on the same day by centrifugation as described in Vigderovich et al. (2022).

**Table 1:** Experiments detail summary.

Experiment	Sediment collection time	Treatment	No. of bottles	^12^CH_4_ [mL]	^13^CH_4_ [mL]	CH_4_ in headspace [%]	Hematite [mM]	O_2_ in headspace [%]	^13^CH_3_OH [mM]	BES [mM]	Sampling point for metagenome/16S rRNA gene [days]	Duration
A	Aug-17	^13^CH_4_ + hematite+1% O_2_	2	1	0.5	7.5	10	1			0, 52	70
		^13^CH_4_ + hematite+0.1% O_2_	2	1	0.5		10	0.1				
		^13^CH_4_ + hematite	2	1	0.5		10					
		killed+^13^CH_4_ + hematite+1% O_2_	1	1	0.5		10	1				
B	Sep-18	^13^CH_4_	2		1	5						111
		^13^CH_4_ + hematite	2		1		10					
		^13^CH_4_ + hematite+BES	2		1		10			20		
		^13^CH_4_ + hematite+0.3% O_2_	2		1		10	0.3				
		^13^CH_4_ + hematite+1% O_2_	2		1		10	1				
		^13^CH_4_ + hematite+20% O_2_	2		1		10	20				
		^13^CH_4_ + hematite+1% O_2_ + BES	2		1		10	1		20		
		Killed+^13^CH_4_ + hematite+1% O_2_ + BES	2		1		10	1		20		
C	Mar-21	CH_4_ + hematite+1% O_2_	5	1.5		4.2	10	1				37
		N_2_ + hematite+1% O_2_	3				10	1				
D	Dec-18	no additions	3								0 (two samples)	147
		^13^CH_3_OH	3						0.56		459	
		^13^CH_4_	3		1	4.2						
		Killed+^13^CH_3_OH	2						0.56			
E	Dec-18	no additions	2								0	129
		^13^CH_3_OH	2						0.56			
		^13^CH_3_OH + BES	2						0.56	20	284	
		^13^CH_3_OH + hematite	2				10		0.56		284	
		hematite	2				10					

### 2.3. Experimental settings

**Figure 1:**
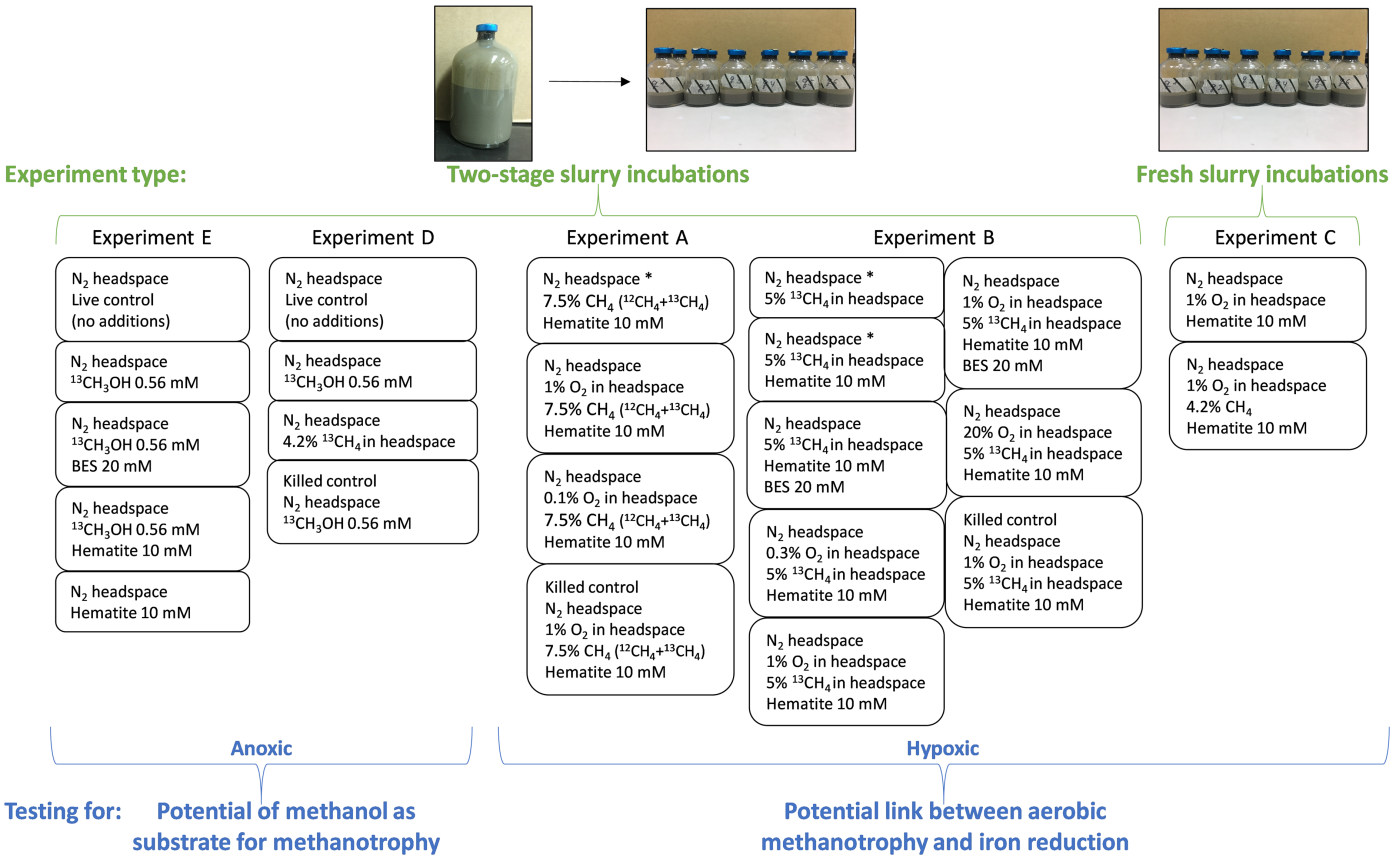
Summary of the experimental settings. Two experiment types are presented (in green): “fresh slurry incubations” with freshly collected methane-generating sediments and porewater from the same depth in a 1:3 sediment-to-porewater ratio. This type includes only experiment C. The second type is “two-stage slurry incubations.” Methane-generating sediments are incubated first in a 1:1 sediment-to-porewater ratio with ^13^C-labeled methane. In the second stage, the slurry is divided into smaller incubations with different amendments and diluted to a 1:3 sediment-to-porewater ratio. This type includes experiments A, B, D, and E. The experiments are divided according to their purpose (in blue). Experiments A–C test the link between aerobic methanotrophy and iron reduction by oxygen injections; thus, they are hypoxic experiments. In these experiments, δ^13^C_DIC_, dissolved Fe(II) and oxygen concentrations in the headspace were measured regularly. A sample from the 1% O_2_ treatment in experiment A was taken for metagenome and lipid analyses. Experiments D and E test methanol as a potential intermediate in methanotrophy under anoxic conditions. In these experiments, δ^13^C_DIC_, dissolved Fe(II) and CH_4_ concentrations were measured. Samples from both experiments were taken for 16S rRNA gene amplicon-based sequencing and lipid analysis. Asterisks denote treatments without oxygen injections (i.e., 0% O_2_ in the headspace).

Five experiments are presented in this study, one of them (experiment C) is a slurry experiment set up with freshly collected sediment and is described below. The other four experiments (A, B, D, and E) are long-term two-stage slurry experiments (details in Vigderovich et al., 2022). In short, sediments from the methane generating zone of the collected cores were transferred within 48 h of their collection, under anaerobic conditions, to a 250 mL pre-autoclaved glass bottle and diluted with porewater extracted from the methane generating sediments to reach a 1:1 sediment-to-porewater ratio (pre-incubated slurry). The incubations were flushed with N_2_ (99.999%, MAXIMA, Israel) and methane (^12^CH_4_ + ^13^CH_4_, 99.99%, MAXIMA, Israel and 99%, Sigma-Aldrich, respectively) was injected into the incubations to reach 20% of the headspace. After at least 3 months of incubation, sub-samples (18–20 g each) from each pre-incubated slurry were transferred under a laminar hood, with continuous flushing of N_2_ to 60 mL pre-autoclaved glass bottles. The slurry was then diluted with filtered (0.22 μm) fresh anoxic porewater from the same depth as the sediments to reach a 1:3 sediment-to-porewater ratio. All experiments were kept in the dark at 20°C. The bottles were shaken before every porewater sampling point, before oxygen measurements, and after every oxygen injection. This was to ensure a homogeneous distribution of the oxygen and the dissolved constituents. We describe below each experiment; details summary of the experiments can be found in Table 1 and Figure 1.

In experiment A, seven sub-samples of pre-incubated (set-up in August 2017) slurry were transferred to seven 60 mL experiment glass bottles. Hematite (Sigma-Aldrich, <5 μm, 99%) was added to six of the bottles to reach final concentration of 10 mM, as was done previously in Vigderovich et al. (2022). Each slurry was further diluted with fresh, filter-sterilized, and anoxic porewater and was crimped-sealed. The final headspace volume in the experiment bottles was 20 mL. The bottles were flushed with N_2_ for 5 min, shaken vigorously, and flushed again thrice (Sivan et al., 2014) to confirm anoxic starting conditions. This was verified with an optical oxygen sensor (details in the analytical methods below). The killed control bottle was autoclaved twice, cooled and only then hematite was added to the killed control bottle. Finally, 1.5 mL of methane was injected into all the experiment bottles (1 mL ^12^CH_4_ + 0.5 mL ^13^CH_4_) to reach final concentration of 7.5% methane in the headspace. The experiment consisted of four treatments, 0% O_2_ + hematite, 1% O_2_ + hematite, 0.1% O_2_ + hematite, and killed control with 1% O_2_ + hematite (“% O_2_” refers to the oxygen concentrations in the headspace). The treatments were set up in duplicates. The duration of this experiment was 70 days. During that time porewater samples were taken for dissolved Fe(II) concentrations and δ^13^C-DIC analyses. Oxygen gas (99.999%, MAXIMA, Israel) was injected into the specific bottles once a week and the oxygen levels in the headspace were monitored. A sample was taken from the 1% O_2_ treatment at the start of the experiment and after 52 days for metagenome analysis. The experiment bottles were kept after the experiment ended, and a sample for lipid analysis was taken after 558 days.

Experiment B was set-up from pre-incubated slurry (set-up in September 2018), similarly to experiment A. It consisted with the seven following treatments ^13^CH_4_, 10 mM hematite, hematite + BES, 0.3% O_2_ + hematite, 1% O_2_ + hematite, 20% O_2_ + hematite, 1% O_2_ + hematite + BES, and killed control + 1% O_2_ + hematite + BES. 1 mL of BES stock solution (0.8 M) was injected into the specific treatment bottles to reach a final concentration of 20 mM, as was previously shown to inhibit the AOM in these sediments (Bar-Or et al., 2017). The final headspace volume of the bottles was 24 mL. The treatments were set up in duplicates. Due to a mistake, ^13^C-labeled methane was injected into all experiment bottles in two pulses, 200 μL in the beginning of the experiment and another 1 mL after 21 days, reaching 5% methane in the headspace. It should be noted that in this experiment (and in experiment D) only ^13^C-labeled methane was injected into the bottles, in a lower volume than experiment A. The methane concentrations were enough to sustain methane oxidation, and the higher labeling resulted in a faster ^13^C-labeling of the DIC in the bottles without oxygen. During the experiment time of 111 days, water samples were taken for dissolved Fe(II) concentrations and δ^13^C_DIC_. In addition, the oxygen levels were monitored in the headspace, and oxygen gas was injected into the specific bottles once a week.

Fresh sediments for experiment C were collected from the methane generating zone of a core collected in March 2021, were cut under anaerobic conditions into a zip lock bag, and 7 g of sediment sample was transferred to eight pre-autoclaved 60 mL glass bottles under a laminar hood. Hematite was added to all bottles (final concentration of 10 mM). Fresh, filter-sterilized, and anoxic porewater from the same depth as the sediments was added to reach a 1:3 sediment-to-porewater ratio. The final headspace volume was 32 mL. The bottles were crimped-sealed and flushed with N_2_ for 1 h and stored in the dark at 4°C for 5 days. To the headspace of each bottle, 1.5 mL of air was injected to reach 1% oxygen in the headspace. 1.5 mL of methane was added to five of the bottles, and 1.5 mL of N_2_ to the rest. Methane concentrations in the bottles headspace were 4.2%. The experiment consisted of two treatments: hematite + O_2_ + CH_4_ in a N_2_ headspace and hematite + O_2_ in a N_2_ headspace. Oxygen concentrations were monitored in the headspace daily and when the levels depleted, the bottles were flushed with N_2_, subsequently, air was reinjected to all bottles, and CH_4_ to the relevant treatment bottles. The experiment bottles were sampled for dissolved Fe(II) concentrations and metagenome analysis (not presented here). The experiment’s duration was 37 days.

Experiment D was set-up from a pre-incubated slurry (set-up in December 2018) in the same fashion as experiment B and consisted of four treatments, ^13^CH_3_OH, ^13^CH_4_, killed control +^13^CH_3_OH, and live control (no additions). For the methanol labeling, a stock of 101 mM concentration of ^13^C-labeled methanol was prepared. Then, 0.2 mL of the stock was injected into the relevant experiment bottles. The final ^13^CH_3_OH concentration in the bottles was 0.56 mM. The killed control bottles were autoclaved twice and cooled, only then ^13^CH_3_OH was injected into them. The final head space volume in the experiment bottles was 24 mL. One milliter of ^13^C-labeled methane was injected into the relevant treatment bottles (4.2% methane in the headspace). All the treatments were set up in triplicates except for the killed control, which was set up in duplicates due to the limited amount of the original 1:1 slurry that was used for this experiment. The duration of this experiment was 147 days, in which porewater was sampled for dissolved Fe(II) concentrations and δ^13^C_DIC_, and the headspace was sampled for methane concentrations and δ^13^C_CH4_. Two samples for 16S rRNA amplicon-based sequencing were taken from the unamended slurry at the beginning of the experiment (as *t_0_*), and another sample, after the experiment ended, at day 459 of incubation from the ^13^CH_3_OH treatment. Samples for lipid analysis from the ^13^CH_4_ and ^13^CH_3_OH treatments were taken after 462 days.

Experiment E was set-up from a pre-incubated slurry (set-up in December 2018) similarly to experiment D and consisted of five treatments, live control (no additions), ^13^CH_3_OH, ^13^CH_3_OH + BES, ^13^CH_3_OH + hematite, and hematite. Each treatment was set up in duplicates. Hematite (Sigma-Aldrich, <5 μm, 99%) was added to reach a final concentration of 10 mM. BES was added to the bottles as in experiment B, and ^13^CH_3_OH was added as in experiment D. The final head space volume in the experiment bottles was 24 mL. All treatments were set up in duplicates. The duration of this experiment was 129 days, in which porewater was sampled for dissolved Fe(II) concentrations and δ^13^C_DIC_, and the headspace was sampled for methane concentrations. Samples for 16S rRNA amplicon-based sequencing were taken from the unamended slurry at the beginning of the experiment and after the experiment ended, at day 284 of incubation, from the ^13^CH_3_OH + BES and ^13^CH_3_OH + hematite treatments. Samples for lipid analysis from the same treatments were taken after 287 days.

### 2.4. Analytical methods

#### 2.4.1. Geochemical measurements

Dissolved Fe(II) samples were analyzed using the ferrozine method (Stookey, 1970) by a Hanon i2 visible spectrophotometer at a 562 nm wavelength with a detection limit of 1 μM. Samples for δ^13^C_DIC_ and δ^13^C_CH4_ values were measured on a DELTA V Advantage Thermo Scientific isotope-ratio mass spectrometer (IRMS) with a precision of ±0.1‰. Reported results refer to the Vienna Pee Dee Belemnite (VPDB) standard. Oxygen concentrations in the headspace were measured by a fiber optic oxygen meter (Fibox 3 trace, PreSens), using an optical oxygen sensor (type PSt6) glued to the inside of the experiment bottle, with a detection limit of 1 ppb. Methane concentrations were measured on a gas chromatograph (FOCUS GC, Thermo Fisher), equipped with a flame ionization detector (FID) with a detection limit of 1 nmol of methane.

#### 2.4.2. Lipid analysis and calculation of new production

A sub-set of samples (Table 2) was investigated for the assimilation of ^13^C-labeled methane into polar lipid-derived fatty acids (PLFAs) and ether lipid-derived hydrocarbons. A total lipid extract (TLE) was obtained according to Sturt et al. (2004) based on a modified Bligh & Dyer protocol. Before extraction, 1 μg each of 1,2-diheneicosanoyl-sn-glycero-3-phosphocholine and 2-methyloctadecanoic acid was added as internal standard. PLFAs in the TLE were converted to fatty acid methyl esters (FAMEs) using saponification with KOH/MeOH and derivatization with BF_3_/MeOH (Elvert et al., 2003). Total ether lipid-derived biomarkers in the TLE were obtained using ether cleavage with BBr_3_ followed by reduction with lithium triethylborohydride, forming hydrocarbons (Lin et al., 2010). For the O_2_-treated sample, ether lipid-derived hydrocarbons were obtained from the intact polar fraction, which was separated from the apolar archaeal lipid compounds using preparative liquid chromatography (Meador et al., 2014) and processed as those present in the TLE. Both FAMEs and ether-cleaved hydrocarbons were analyzed by a GC–mass spectrometry (GC–MS; Thermo Finnigan TRACE GC coupled to a TRACE MS) for identification and by GC-IRMS (Thermo Scientific TRACE GC coupled via a GC IsoLink interface to a DELTA V Plus) to determine δ^13^C values using column and temperature program settings described by Aepfler et al. (2019). δ^13^C values are reported with an analytical precision better than 1‰ as determined by long-term measurements of an *n*-alkane standard with known isotopic composition of each compound. The incorporation of ^13^C-methane or ^13^C-methanol into PLFAs was calculated as the product of excess ^13^C and the amount of PLFA carbon based on quantification via GC-FID measurements. Excess ^13^C is the difference between the fractional abundance (F) of ^13^C in PLFAs after relative to the *t_0_* sample where *F* = ^13^C/(^13^C + ^12^C) = *R*/(*R* + 1), with R being derived from the measured δ^13^C values as *R* = (δ^13^C/1000 + 1) × RVPDB.

**Table 2:** Isotope change (in ‰) of most diagnostic bacterial fatty acids and archaeol-derived phytane compared to DIC as an overall indicator of methane/methanol turnover (complete isotopic pattern can be found in Supplementary Table S5).

Experiment	Treatment	iC_15:0_	C_16:1ω7_	Phytane	δ^13^C_DIC_	Sum of ^13^C in bacterial fatty acids (ng ^13^C/g dw)
D	^13^CH_4_	−27	−42	−3	300	1.2
D	^13^CH_3_OH	170	−7	2,300	2,500	7.8
E	^13^CH_3_OH + BES	710	850	−5	2,500	20.3
E	^13^CH_3_OH + hematite	710	140	1,600	2,800	16.1
A	^13^CH_4_ + hematite+1% O_2_	480	4,100	16	2,500	57.9

In addition, the calculated sum of newly produced bacterial fatty acids is presented. Samples were taken from experiments D and E after 462 and 287 days of incubation, respectively.

#### 2.4.3. DNA extraction, 16S rRNA gene V4 amplicon-sequencing, and metagenomics

DNA was extracted from six sediment slurries samples (stored at −20°C) of the experiments D and E. Two samples from t_0_ of experiment D and one sample from the ^13^CH_3_OH treatment. From experiment E one sample of t_0_ and one sample from each of the following treatments ^13^CH_3_OH + BES and ^13^CH_3_OH + hematite. This was done by using the PowerSoil™ DNA Isolation Kit (QIAGEN, Hilden, Germany), according to the manufacturer’s instructions and stored at −80°C. The 16S rRNA gene amplicon-based sequencing targeting the V4 region was performed using modified primer pair with consensus sequence CS1_515F (ACACTGACGACATGGTTCTA CAGTGCCAGCMGCCGCGGTAA) and CS2_806R (TACGGT AGCAGAGACTTGGTCTGG ACTACHVGGGTWTCTAAT; Sigma-Aldridge, Israel; Walters et al., 2015). 25 μL reactions of the first PCR contained 12.5 μL KAPA HiFi HotStart ReadyMix (KAPA Biosystems, Wilmington, WA, United States) and 0.75 μL forward and reverse primers at a final concentration of 300 nM each. The PCR conditions were an initial denaturation at 95°C for 3 min, followed by 25 cycles of 98°C for 20 s, 60°C for 15 s, and 72°C for 30 s. PCR products were visualized on a 2% agarose gel to measure the bands’ relative intensity. Samples were pooled and purified using calibrated Ampure XP beads and used for library preparation. PCR visualization, purification, library preparation, and sequencing (2 × 250 bp pair-end reads) were performed at HyLabs (Israel) and sequenced on an Illumina MiSeq platform. Metagenomic libraries were constructed using NEBNext® Ultra™ IIDNA Library Prep Kit (Cat No. E7645) and sequenced as *circa* 100 million 150 bp paired-end reads using the Illumina NovaSeq at Novogene (Singapore). For metagenomics, we used the genomic DNA from two samples of the 1% O_2_ + hematite treatment of experiment A: (i) at beginning of the experiment (t0) and (ii) after 52 days. Total genomic DNA was extracted using the DNeasy PowerLyzer PowerSoil Kit (Qiagen). Genomic DNA was eluted using 50 μL of elution buffer and stored at −20°C. Metagenomics libraries were prepared at the sequencing core facility at the University of Illinois Chicago using the Nextera XT DNA library preparation kit (Illumina, United States). Between 19 and 40 million, 2 × 150 bp paired-end reads per library were sequenced using Illumina NextSeq 500 metagenomes.

#### 2.4.4. Bioinformatics

For the amplicon reads, QIIME2 V.2020–11 (Bolyen et al., 2019) was used for demultiplexing of the paired-end reads and following analysis. Sequence quality was assessed using the q2-demux plugin. Merging of reads into Amplicon Sequence Variants (ASVs) was done with DADA2 (Callahan et al., 2016), using the q2-dada2 plugin. To account for length variations, ASVs were defined by clustering at 100% similarity (Rognes et al., 2016). The 138-SILVA QIIME-release database was used for taxonomy assignment, at 99% clustering (Quast et al., 2012). The q2-feature-classifier plugin (Bokulich et al., 2018) was used to build the classifier (Extract-reads and fit-classifier-naive-bayes methods). Classification of the ASVs was done via the classify-sklearn method (ver. 0.23.1; Pedregosa et al., 2011). Downstream analysis in R was performed using the packages phyloseq (McMurdie and Holmes, 2013), and ggplot2 (Wickham, 2016). The QIIME2 feature-table plugin was used to generate the Heatmaps (Hunter, 2007; McDonald et al., 2012).

For metagenomics, read quality control, assembly, and binning were performed within ATLAS v2.1 framework (Kieser et al., 2020), using SPAdes v3.14 (Prjibelski et al., 2020) as assembler, as well as binning using metabat (Kang et al., 2015) and maxbin2 (Wu et al., 2016), finalized by DAStool (Sieber et al., 2018). Metagenome-assembled genomes were dereplicated using the 0.975 cutoffs with dRep (Olm et al., 2017). The relative read abundance was estimated by mapping the metagenomic reads at 0.9 identities to the genomes using BBMap (Bushnell, B., https://sourceforge.net/projects/bbmap/).

### 2.5. Statistical analysis

We measured the change in Fe(II) concentrations after the addition of methane or nitrogen in experiment C in four time points (after 0, 11, 23, and 37 days). To assess the change over time, we performed a separate statistical analysis for both treatments, via one-way repeated measures ANOVA. To analyze the differences between treatments, Two-way repeated measures ANOVA was performed. *Post hoc* tests were performed by pairwise *t*-tests, with Bonferroni correction for multiple testing. To achieve normality Fe(II) concentrations were Log10 transformed. Analysis was performed in R using the rstatix package.

## 3. Results

### 3.1. Aerobic methanotrophy and iron reduction

**Figure 2:**
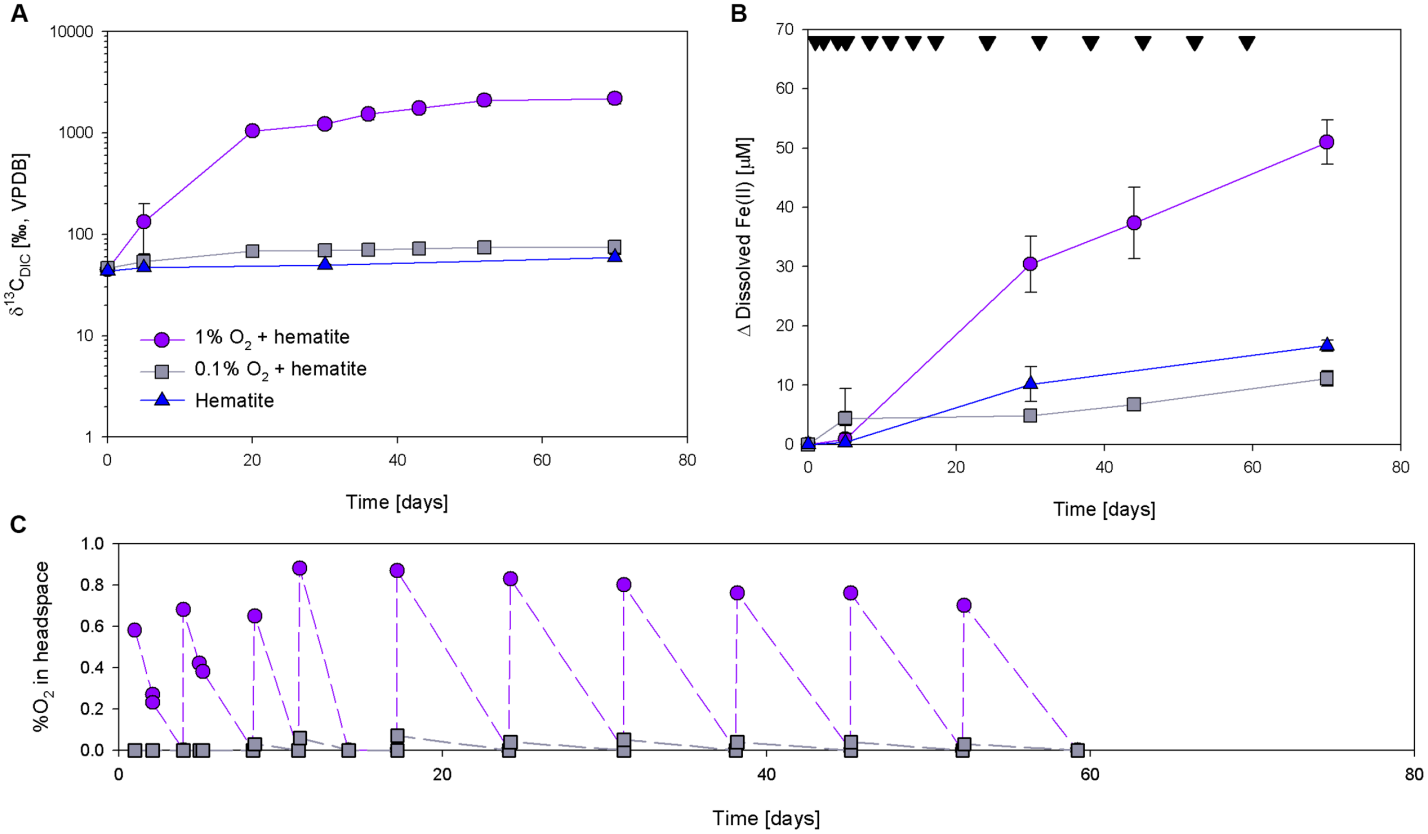
Development of δ^13^C_DIC_ **(A)**, Δ dissolved Fe(II) **(B)**, and %O_2_ **(C)** during experiment A with the additions of ^13^C-labeled methane, 10 mM hematite and injection of oxygen. Black upside-down triangles in panel **(B)** represent oxygen injections time. Error bars represent the average deviation from the mean of duplicate bottles.

Three incubation experiments (A, B, and C) tested how exposure to limited amounts of oxygen affects the methanotrophy and iron reduction in methane-generating sediments of Lake Kinneret. In experiment A, oxygen was injected repeatedly to reach two final concentrations (1 and 0.1%) in the headspace. The δ^13^C_DIC_ values of the 1% O_2_ treatment increased intensively and reached up to 2,500‰ (Figure 2A; Supplementary Table S1). In the 0.1% treatment, the values increased by 37‰ during the experiment. The values of the bottles without the addition of oxygen also increased during the experiment, but only by 19‰. The average initial dissolved Fe(II) concentrations were 20 ± 8 μM. The highest Fe(II) concentrations change was in the 1% O_2_ treatment (Figure 2B; Supplementary Table S2). The change in Fe(II) concentrations of the control and the 0.1% O_2_ treatments were 16 and 11 μM, respectively. Using the more frequent O_2_ measurements in the beginning of the experiment, the oxygen consumption rate was calculated to be 0.03% O_2_ per g sediment per day (Figure 2C).

**Figure 3:**
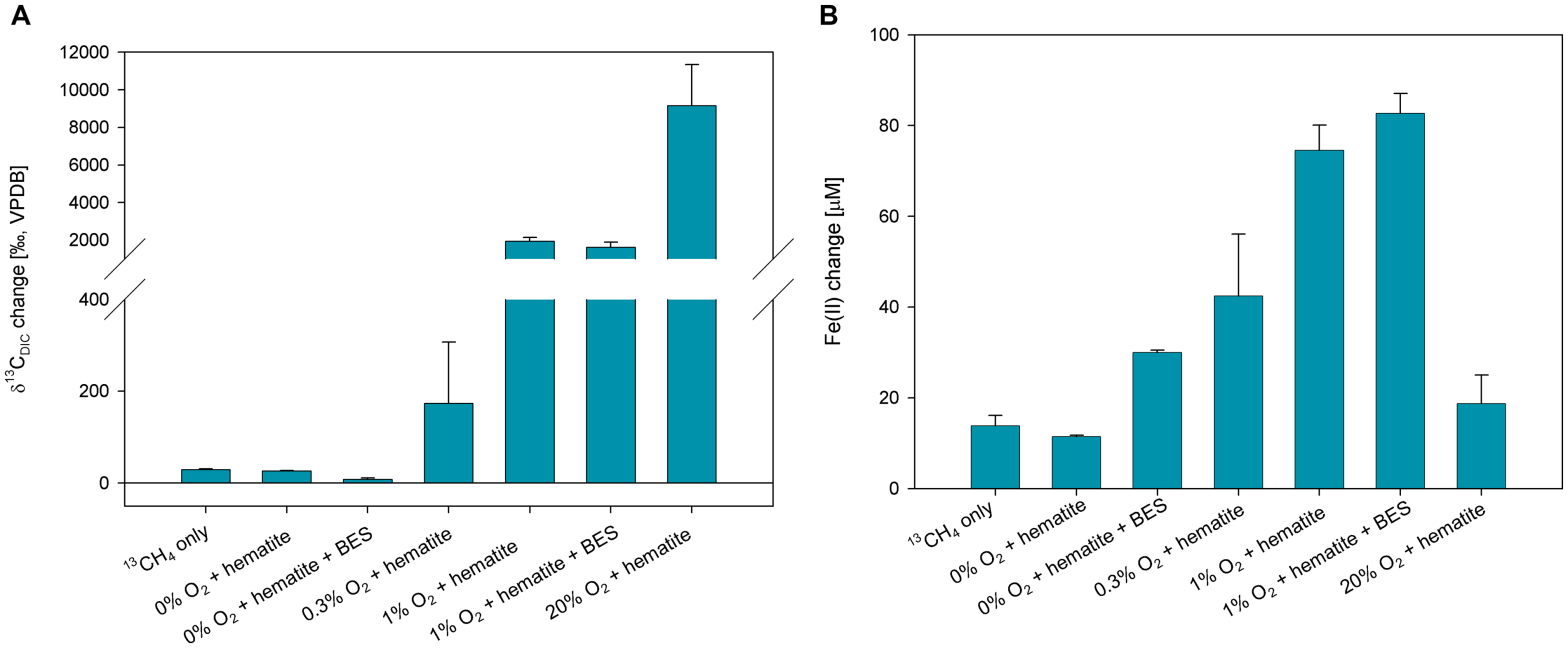
Net change of δ^13^C_DIC_ after 61 days **(A)** and dissolved Fe(II) after 111 days **(B)** of experiment B, where 10 mM hematite and 20 mM BES were added in addition to ^13^C-labeled methane and oxygen injections. Error bars represent the average deviation from the mean of the duplicate bottles.

To corroborate the findings obtained in experiment A and to extend our comprehension of the impact of oxygen on iron reduction, experiment B (Figure 3; Supplementary Figure S1) was set up. Oxygen was injected to reach three different concentrations of 0.3, 1, and 20% in the headspace. The δ^13^C_DIC_ values of the 20% O_2_ treatment were the highest (>10,000‰), and the 1% O_2_ treatments, with and without BES, reached approximately 2,000‰ after 60 days (Figure 3A; Supplementary Table S3). Both treatments were not measured for δ^13^C_DIC_ after 60 days because of a memory effect of the IRMS (due to the very high isotopic values). The δ^13^C_DIC_ values of the 0.3% oxygen treatment reached 2,381‰ at the end of the experiment. The isotopic values of the ^13^CH_4_-only and hematite treatments increased as well and reached 261 and 210‰ (respectively) by the end of the experiment. The δ^13^C_DIC_ values of the hematite and BES treatment did not change throughout the experiment (Supplementary Figure S1). The average initial dissolved Fe(II) concentrations were 40 ± 6 μM. The change in the Fe(II) concentrations increased in all treatments, except for the 20% O_2_ treatment which remained the same with low concentrations (Figure 3B; Supplementary Table S4). The highest change was in the 1% O_2_ treatment with the addition of BES, then the treatment with 1% O_2_ without BES, then 0.3% O_2_ treatment. Dissolved Fe(II) concentrations of the treatments without oxygen increased as well, however much lower than the treatments to which oxygen was injected.

**Figure 4:**
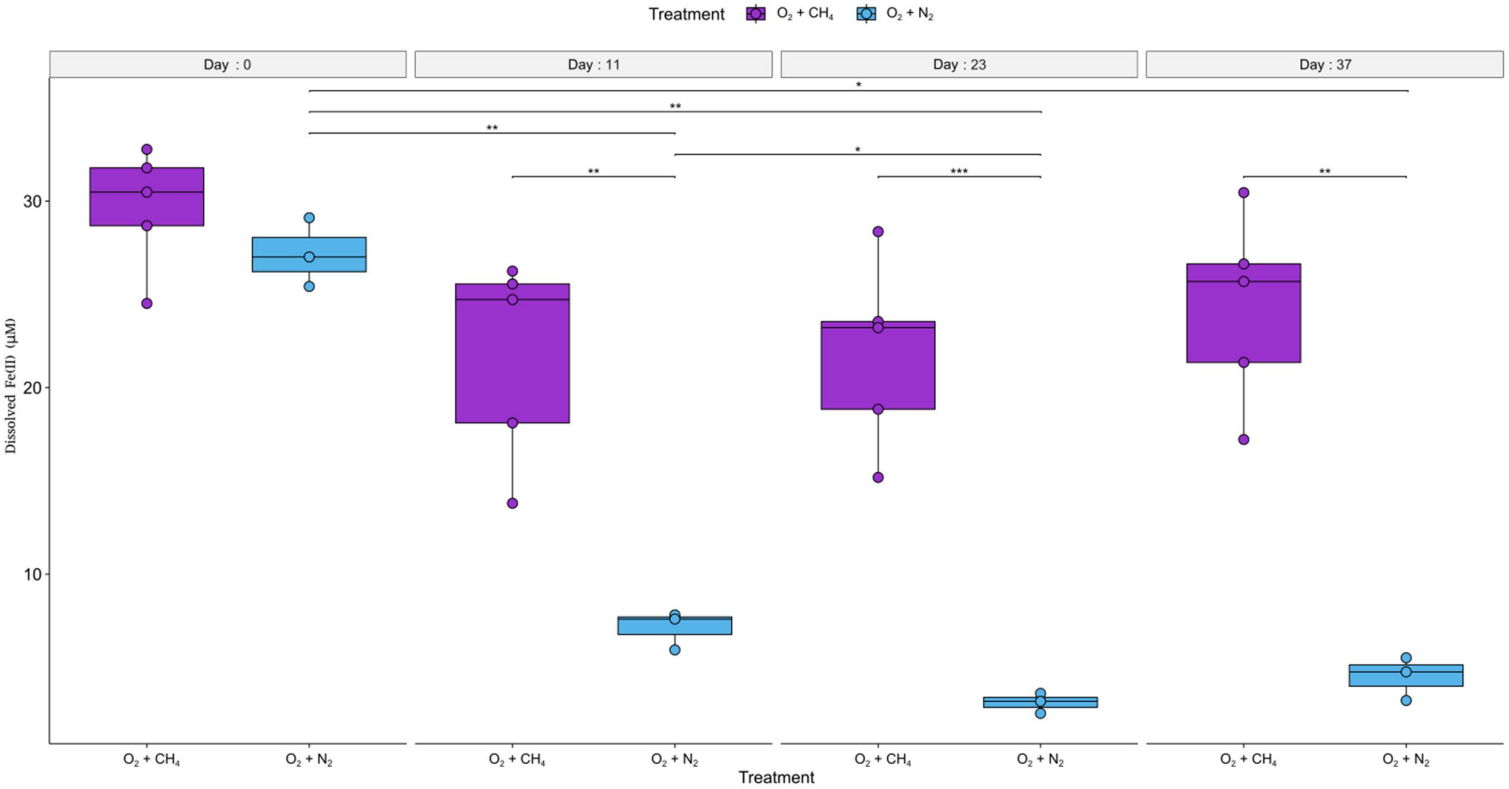
Development of dissolved Fe(II) concentrations over time in experiment C are presented as boxplots, with (purple) and without (cyan) methane in the headspace. The error bars indicate the measurements of replicate bottles. Significance levels; ^*^p ≤ 0.05; ^**^p ≤ 0.01; ^***^p ≤ 0.001. Air was injected into the bottles to reach 1% O_2_ in the headspace at the following time points: 2, 5, 10, 11, 12, 13, 14, 15, 18, 21, and 23 (days from the start of the experiment).

The third experiment (experiment C) used fresh sediments and consisted of two treatments, one with a headspace of N_2_ and methane and the other with a headspace of only N_2_. Air was injected into both treatments to reach a 1% O_2_ concentration in the headspace and reinjected each time the oxygen depleted. The average initial dissolved Fe(II) concentrations were 27 ± 3 μM. Fe(II) concentrations measured in the treatment with methane addition did not alter significantly throughout the experiment. A slight non-significant average decrease (of 8 μM) was noted between day 0 and day 11. Final concentrations after 37 days were 24.3 ± 5 μM. In the treatment without methane, concentrations decreased significantly (*F* = 122.1, *p* = 9.1×10^−6^). Fe concentrations were lower at the end of the experiment after 37 days by 22.7 μM (*t* = 11.1, *p* = 0.032) and stood at 4.5 ± 1.2 μM. Similarly, *post hoc* pairwise comparisons indicate concentrations were higher at day 0, compared to the other time points (Figure 4; Supplementary Data). Two-way repeated measured ANOVA showed a significant correlation between time and treatment (*F* = 73.7, *p* = 4 × 10^−5^). *Post hoc* analysis revealed Fe(II) concentrations did not differ at day 0. Subsequently, at the following time points (11, 23, and 37 days), concentrations were significantly and consistently lower at the treatment without methane (Figure 4; Supplementary Data).

**Figure 5:**
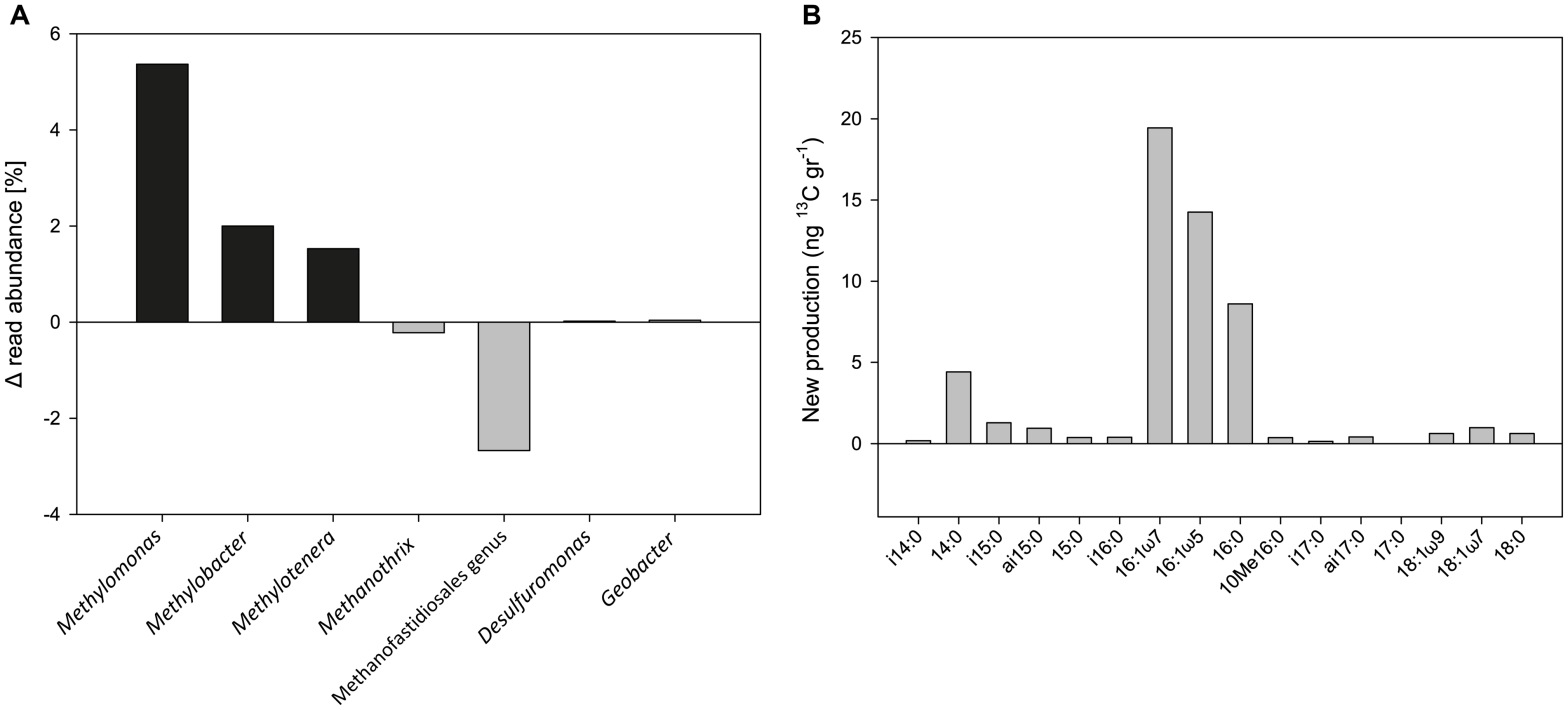
**(A)** Change in the relative abundance of the 16S rRNA gene from metagenome analysis of the 1% O_2_ with hematite addition treatment of Experiment A (sampled after 52 days). **(B)** Newly produced fatty acids of the same treatment (in ng ^13^C/g dw) after 558 days.

Metagenomic analysis was performed on the 1% O_2_ + hematite treatment at t_0_ and after 52 days. The results show an increase in the relative abundance of the methanotrophic bacteria *Methylomonas* (5.4%), *Methylobacter* (2%), and the methylotrophic bacterium *Methylotenera* (1.5%) during the experiment (Figure 5A). A small increase was also observed in the relative abundance of *Desulfuromonas* (0.02%) and *Geobacter* (0.04%). In this treatment, a decrease of 0.2 and 2.7% in the relative abundance of archaea *Methanothrix* and the Methanofastidiosales order (respectively) was observed.

Isotopes and concentrations analyses of PLFAs during the oxygen experiment provide a base for calculating the production of newly formed fatty acids. Our analysis shows a pattern indicative of aerobic methanotrophs being highly stimulated (Figure 5B), with new ^13^C production ranging between 4 and 20 ng ^13^C/g dw observed for dominating C_14:0_, C_16:1ω7_, C_16:1ω5_, and C_16:0_. This PLFA pattern matches the one provided by Bar-Or et al. (2017) (Supplementary material), but the new production is four times higher during active addition of oxygen_,_ as performed here.

### 3.2. Methanol intermediate as a potential substrate for methanotrophy

**Figure 6:**
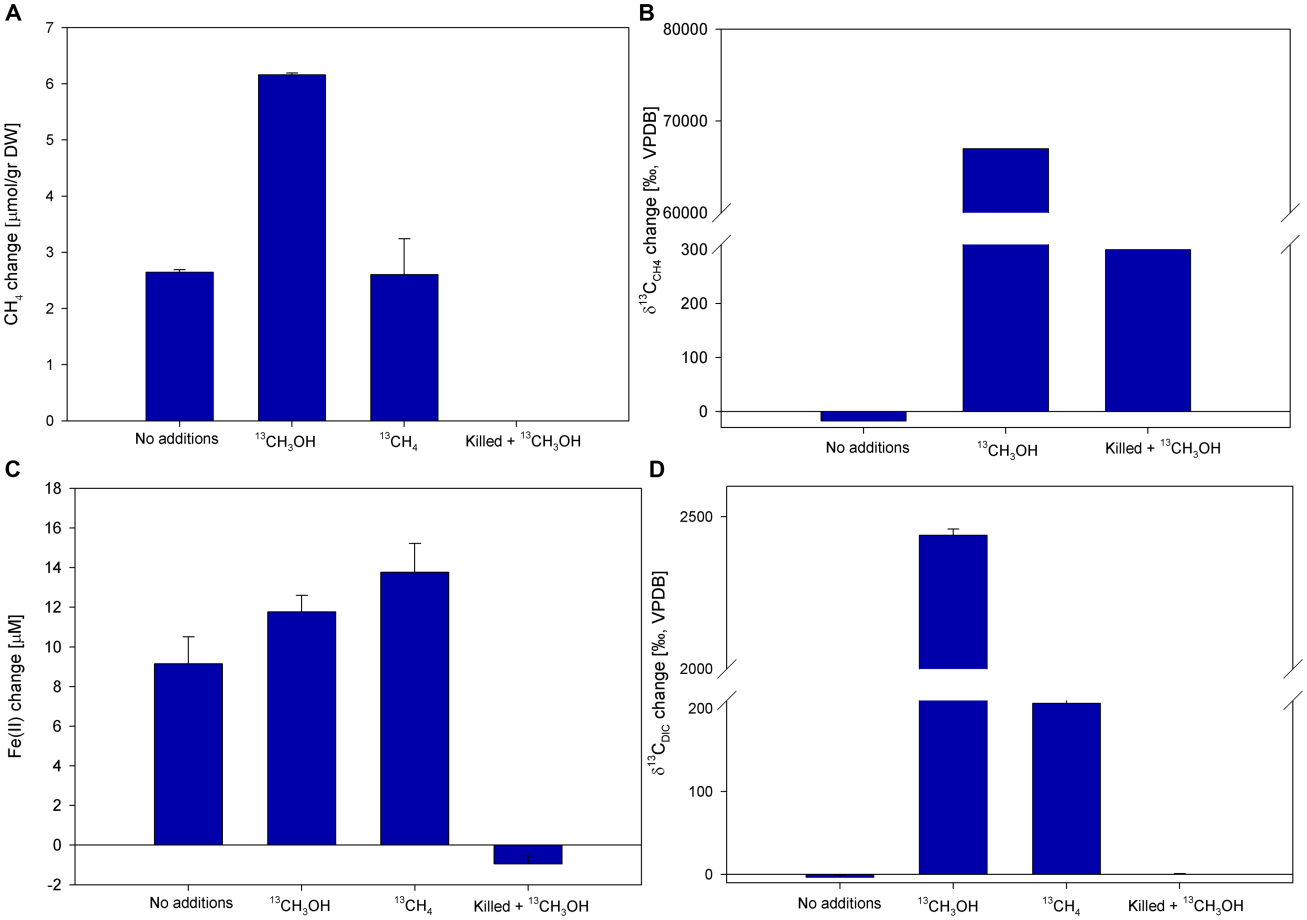
Net changes of CH^4^ **(A)**, δ^13^C_CH4_ **(B)**, dissolved Fe(II) **(C)**, and δ^13^C_CH4_ **(D)** after 147 days of Experiment D with the addition of ^13^C-labeled methanol (δ^13^C_CH4_ was measured after 61 days). Error bars represent the average deviation from the mean of triplicate bottles.

We explored the involvement of methanol in methanotrophy and iron reduction, due to our former suggestion of potential methane activation by archaea and the release of available intermediates to the methanotrophs (Bar-Or et al., 2017). Of those, methanol would be the most probable candidate. During those anoxic slurry experiments, we observed an inhibition of the overall process by BES addition. Using ^13^C-labeled methanol, two incubation experiments (D and E) tested the involvement of methanol. In experiment D (Figure 6; Supplementary Figure S2), the methane concentrations increased in all treatments except for the killed control. The highest change in methane concentrations was observed in the methanol treatment (6.2 μmol/g dw). The change in methane concentrations was the same in the no-addition and the methane treatments (2.6 μmol/g dw; Figure 6A). The δ^13^C_CH4_ values in the ^13^C-labeled methanol treatment reached the highest value (67,000‰) compared to the no-addition value (−18‰) and the killed control (300‰; Figure 6B). Throughout the experiment δ^13^C_DIC_ values of the ^13^C-labeled methanol treatment increased to ~2,500‰ in, and to 260‰ in the ^13^C-labeled methane treatment (Figure 6D). The average initial dissolved Fe(II) concentrations were 28 ± 6 μM. The dissolved Fe(II) concentrations increased in all treatments except for the killed control treatment, with the highest increase noted in the ^13^C-labeled methane treatment (13.8 μM; Figure 6C).

**Figure 7:**
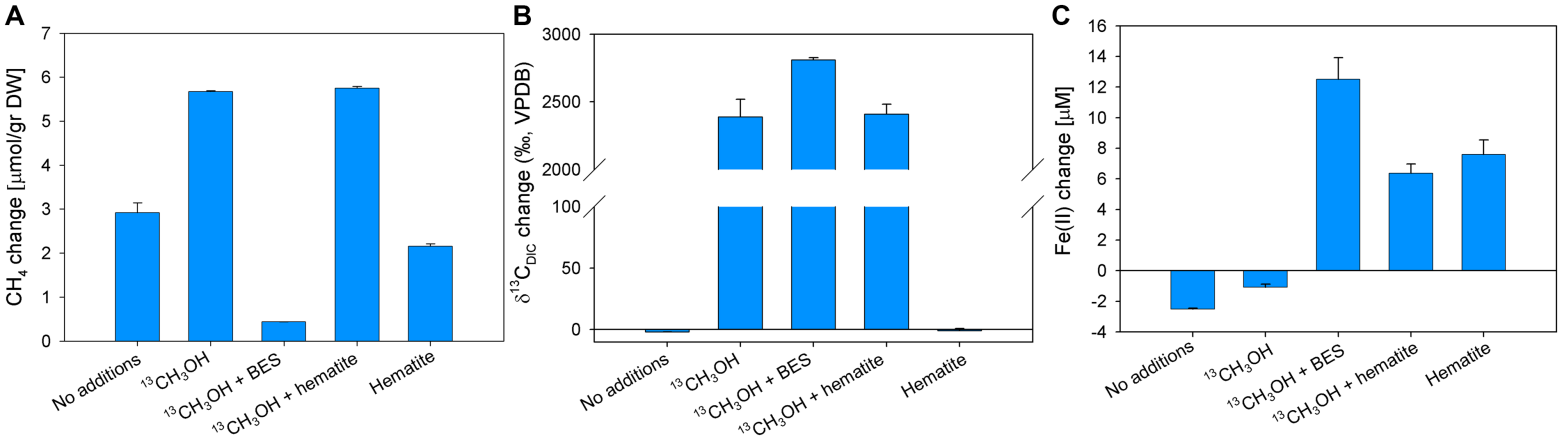
Net changes of CH_4_ **(A)**, δ^13^C_DIC_ **(B)**, and dissolved Fe(II) **(C)** after 129 days of Experiment E with the addition of ^13^C-labeled methanol, hematite, and BES. Error bars represent the average deviation from the mean of duplicate bottles.

In experiment E, BES and hematite were added to the slurries in addition to ^13^C-labeled methanol to test the involvement of methanogens. Methane concentrations increased in all treatments except for the methanol + BES treatment. The methanol and the methanol + hematite treatments show the highest change of 5.7 μmol/g dw, while the no-addition and hematite treatments increased by 3 and 2 μmol/g, respectively (Figure 7A). The δ^13^C_DIC_ values in the methanol and the methanol + hematite treatments increased during the experiment by 2,389 and 2,409‰, respectively (Figure 7B). The δ^13^C_DIC_ of the methanol + BES increased by the end of the experiment by 2,809‰; however, the slope during the first 14 days was relatively low compared to the slope between day 14 and the end of the experiment (Supplementary Figure S3). The average initial dissolved Fe(II) concentrations were 50 ± 7 μM. Dissolved Fe(II) concentrations increased by about 7 μM in the hematite and methanol + hematite treatments (Figure 7C). In the methanol + BES treatment, the concentrations increased by 12 μM throughout the experiment. The concentrations of the no-addition and methanol treatments remained the same.

Samples from experiments D and E were analyzed for the isotopic composition of bacterial fatty acids and archaeal-derived isoprenoid hydrocarbons (Table 2; Supplementary Table S5). The δ^13^C values of the archaeol-derived phytane in the ^13^C-methanol treatments with and without hematite addition were 1,600 and 2,300‰, respectively. In contrast, in treatments with methane and ^13^C-methanol plus BES δ^13^C values were − 3 and − 5‰, respectively. Fatty acids mostly indicative of heterotrophic bacteria (iC_15:0_, Aepfler et al., 2019) from the ^13^C-labeled methanol treatment were slightly enriched in ^13^C. The enrichment was more pronounced in the treatments with the addition of BES and hematite. In addition, there was a strong ^13^C-enrichment in the fatty acid C_16:1ω7_, which can be affiliated with methylotrophic bacteria (Guckert et al., 1991), specifically considering the conditions applied here. The highest value was found in the treatment with BES (up to 850‰), then with hematite, and a small enrichment in the treatment with methanol only. No change in δ^13^C values of fatty acids was observed in the methane treatment. A combination of fatty acid carbon isotope values and their corresponding concentrations were used to calculate the portion of newly produced fatty acids (in ng ^13^C/g dw; Supplementary Figure S7). These patterns are different from that observed for the oxygen experiment which is highly specific for aerobic methanotrophs and shows much higher ^13^C incorporation. Nonetheless, new production is still fairly high in the methanol treatments with new production ranging from 0.5 to 4.5 ng ^13^C/g dw for the most diagnostic fatty acids iC_15:0_ and C_16:1ω7_. Other fatty acids such as aiC_15:0_, C_16:0_, and C_18:1ω7_ are also showing new production values higher than 1.0 ng ^13^C/g dw, especially when methanogens were inhibited or when iron reduction was stimulated by hematite additions. This trend is also visible using the sum of all newly produced bacterial fatty acids as an indicator of the overall turnover capacity of bacteria during the different treatments (Table 2). The highest amount of new production was found when oxygen is introduced (57.9 ng ^13^C/g dw), whereas the lowest amount was detected during addition of ^13^CH_4_ (1.2 ng ^13^C/g dw). Methanol additions resulted in new productions of 7.8 ng ^13^C/g dw, and 20.3 and 16.1 ng ^13^C/g dw, when BES and hematite was used, respectively.

To study the microbial community associated with methanotrophy related to methanol addition, samples supplemented with methanol (experiment D), methanol and BES, and methanol and hematite (experiment E) were sent for 16S rRNA amplicon-based sequencing. Following taxonomic classification, our analysis indicated archaea accounted for a significant percentage of the microbial population of all treatments (between 28.3 and 37.5%, Supplementary File 1), and the remaining reads were assigned to bacteria. All ASVs of both experiments are also presented in Supplementary File 1. A substantially larger number of Bacterial Classes and Orders was noted (experiments D: 129 classes and 228 Orders; experiment E: 112 classes and 199 Orders), compared to Archaea (experiment D: 17 classes and 24 Orders; experiment E: 18 classes and 28 Orders). Supplementary Figure S4 presents the most abundant (>1%) Bacterial Classes and Archaeal Orders in experiments D and E. Six prominent bacterial Classes accounted for about 50% of the bacterial reads, with slight alteration observed from T0 to the methanol, methanol + BES and methanol + hematite additions: Anaerolineae, Dehalococcoidia, Gammaproteobacteria, Sva0485, Thermodesulfovibrionia, and Aminicenantia (Supplementary Figure S4B). Similarly, the six prominent Archaea orders, accounted for over 80% of the archaeal reads, with minor variations: Bathyarchaeia, Methanomicrobiales, Marine Benthic Group D and DHVEG-1, Woesearchaeales, Methanosarciniales and Methanofastidiosales (Supplementary Figure S4A).

We identified methanogens of the orders Methanosarciniales, Methanomassiliicoccales, Methanomicrobiales, Methanocellales, *Ca.* Methanomethyliales and *Ca.* Methanofastidiosales. Methanogens of the genus *Methanomethylovorans* (0.6% of total reads), were only detected after methanol addition to the slurries, compared to t_0_ of experiment D (Supplementary Figures S5A, S6A). Similarly, the genus *Methanosarcina* (2.4% of total reads) was predominantly found after methanol addition. An increase in the relative abundance of *Ca.* Methanomethylicus was noted (from 0.16 to 0.32% of total reads). In contrast, the relative abundance of the *Methanomassiliicoccaceae* family was reduced (from 0.38 to 0.28% of total reads) in the presence of methanol. Slight decreases were observed after methanol addition in the genera *Methanosaeta* (2.6–1.9% of total reads), *Methanoregula* (1.36–1.5% of total reads), and *Methanolinea* (3.2–2.75% of total reads). As expected, after BES addition, the relative abundance of all the methanogens decreased compared to t_0_ of experiment E, excluding *Methanosarcina* which was only observed after BES addition (Supplementary File 1).

Aerobic methanotrophs were also observed in both experiments (Supplementary Figures S5B, S6B). The Gammaproteobacteria family *Methylophilaceae*, comprising type I aerobic methanotrophs (Deng et al., 2019), was detected at low relative abundance (0.02 and 0.05% of total reads) after the addition of methanol. The family *Methylococcaceae* (class Gammaproteobacteria), which is also comprised of type I methanotrophs (Taubert et al., 2019), was found in all samples of both experiments. After methanol addition, *Methylococcaceae* relative abundance decreased (from 0.28 to 0.21% of total reads). An increase (from 0.2 to 0.3%) was noted after the addition of hematite. *Methylocystis* (Alphaproteobacteria), a type II strictly aerobic methanotroph (Bowman, 2006; Belova et al., 2011), was observed in both experiments at low relative abundance (between 0.04 and 0.08% of total reads). Although *Methylocystis* relative abundance did not change significantly after methanol addition, when methanol and hematite were added, it increased compared to t_0_ (from 0.05 to 0.08% of total reads). Methanol addition also increased the relative abundance of bacteria capable of iron-reduction: *ca. Omnitrophus* (from 1.1 to 1.64% of total reads), *Anaeromyxobacter* (from 0.34 to 0.47% of total reads), *Desulfuromonas* (from 0.07 to 0.15% of total reads) and *Thermoanaerobaculum* (from 1.44 to 1.57% of total reads). Even more profound increases were observed for most of the aforementioned iron reducers in the presence of hematite (Supplementary Figures S5C, S6C, Supplementary File 1).

## 4. Discussion

### 4.1. Aerobic conditions (re)activate methanotrophy and promote net iron reduction

Methane oxidation in Lake Kinneret sediments has been observed in pore-water profiles, models, on-top core, and incubation experiments; however, the observed oxidation was considered anaerobic due to the anoxic nature of the sediments (Adler et al., 2011; Sivan et al., 2011; Bar-Or et al., 2017; Vigderovich et al., 2022). Nevertheless, evidence for aerobic methanotrophy was presented in different microbial profiles (Bar-Or et al., 2015) and incubation experiments (Bar-Or et al., 2017; Elul et al., 2021) of Lake Kinneret sediments and, similarly, in other highly reduced freshwater environments around the world (Blees et al., 2014; Milucka et al., 2015; Oswald et al., 2016b; Martinez-Cruz et al., 2017; Van Grinsven et al., 2020, 2021; Su et al., 2022). This may be due to the slow release of remnant oxygen from clay-containing sediments, as proposed in Wang et al. (2018), or due to the continuous production of low oxygen levels in the anoxic environment, which is immediately used by methanotrophs and thus does not poison the anaerobes (Ettwig et al., 2010; Dershwitz et al., 2021; Kraft et al., 2022). An alternative explanation may be the potential survival of methanotrophs performing anaerobic metabolism under hypoxia conditions (Kits et al., 2015; Orata et al., 2018; Zheng et al., 2020; Cheng et al., 2022). In Lake Kinneret, aerobic methanotrophs and Fe-AOM co-occur at the same depth, raising the possibility that iron reduction is somehow associated with aerobic activity. Here we explored the potential aerobic methanotrophy in hypoxic methane-generating sediments of Lake Kinneret and assessed its influence on iron reduction.

The link between aerobic methanotrophy and iron reduction was investigated by repeated oxygen injections into three sets of initially anoxic slurry incubation experiments with/out of methane in the headspace and with/out inhibition of methanogenesis. Our results indicate first that the aerobic methanotrophs in these sediments can be activated by even small oxygen levels, which means that they are in a dormant-like state or possess the ability to survive under anoxic conditions by using other electron acceptors as was previously shown with related methanotrophs (Kits et al., 2015; Orata et al., 2018; Zheng et al., 2020; Cheng et al., 2021).

Second, interestingly, the net iron reduction increased along with the oxygen levels, up to 1% O_2_ treatment. This increase was unexpected since dissolved Fe(II) was thought to be oxidized quickly when oxygen is introduced to the system, as can be seen in the 20% O_2_ treatment (Figure 3). Generally, Fe(III) reduction is considered to occur mainly under anoxic conditions, where it acts as an electron acceptor instead of oxygen, and the produced Fe(II) is stable (Straub et al., 2001; Lovley et al., 2004). Nevertheless, iron reduction under aerobic conditions has recently been demonstrated in pure culture of iron reducers (Zhang et al., 2019). Zhang et al. (2019) also noted a delay in Fe(II) oxidation under oxic conditions due to metabolites (citric and gluconic acids) and self-produced-siderophores secreted by the specific iron reducers (Actinobacteria) that were tested. These metabolites can bind Fe(II) to form stable complexes. In our slurries, a net increase in Fe(II) was observed in all the treatments where oxygen was injected. Higher levels of injected oxygen (up to 1%) resulted in higher Fe(II) levels. In experiment C, where fresh sediments were incubated (Figure 4), it seems that there is no Fe(II) build-up without the presence of methane, suggesting that methanotrophy is required to explain the increase in net iron reduction. This implies that the methanotrophs/methylotrophs contribute to the observed iron reduction, perhaps by secreting metabolites that are actively used by iron reducers (as a carbon source, for instance) or by functioning as Fe(II)-binding ligands.

The iron reduction in the methane-generating sediments could be performed by iron-reducing bacteria, but also by methanogens (Sivan et al., 2016) and even by aerobic methanotrophs (Zheng et al., 2020); all three have been found in this depth of Lake Kinneret sediments (Bar-Or et al., 2015). It seems that before oxygen injection, natural iron reduction occurs in anoxic sediments by different iron reducers and perhaps by methanogens (Elul et al., 2021). When oxygen is injected, it is used for aerobic methanotrophy and biotic/abiotic Fe(II) oxidation. As a result, new and highly reactive (less crystalline) iron oxides precipitate. The increase in reactive iron oxides encourages iron reduction when oxygen levels are low and net iron reduction increases. In addition, some of the aerobic methanotrophs (i.e., *Methylomonas* and *Methylosinus* species) are known to be able to switch to iron reduction metabolism when oxygen levels are low (Zheng et al., 2020), this contributes to the increase in net Fe(II) concentrations. Experiment C indicates that when methanotrophy is scarce, there is no increase in the net iron reduction. This suggests that dissolved Fe(II) does not only increase due to the presence of oxygen but that methanotrophic bacteria activity is necessary for Fe(II) to accumulate, as was shown by Zhang et al. (2019). This potentially encourages iron reduction due to metabolites or an intermediate release during methanotrophy.

Hematite is considered a less reactive and more stable iron oxide compared to non-crystalline Fe-oxides, such as ferrihydrite, goethite or other iron(hydr)oxide (Poulton et al., 2004). Here, hematite was chosen to be the Fe(III)-oxide added to the slurries due to its stable nature, so that it will not disturb the system and shift it toward iron cycling. In addition, it is found naturally in the sediments and was shown to be the most available iron oxide for Fe-coupled AOM in incubations with Lake Kinneret sediments (Bar-Or et al., 2017). Here, in the hypoxic incubations, where oxygen was injected regularly, an aerobic metabolic pathway for methanotrophy was observed that promoted the iron reduction. It could be that addition of more reactive iron oxide to the experiments would cause more intense iron reduction.

From the results of the metagenome analysis on the 1% O_2_ treatment of experiment A, it is evident that the methane oxidation is most likely performed by the type I methanotrophs *Methylomonas* and *Methylobacter* (Figure 4A). This is in line with previous observations from Lake Kinneret sediments, where the Methylococales order was detected in a microbial sediment profile and a slurry incubation experiment (Bar-Or et al., 2015, 2017; Elul et al., 2021). These methanotrophs were also noted in other anoxic/hypoxic freshwater environments (Martinez-Cruz et al., 2017; Cabrol et al., 2020; Van Grinsven et al., 2020, 2021; Su et al., 2022). Another aerobic bacterium detected in experiment A is the non-methane-oxidizing methylotroph *Methylotenera*. This methylotroph is known to co-occur with both *Methylomonas* and *Methylobacter* and oxidizes methanol excreted by the latter two as an intermediate during the methanotrophy process (Beck et al., 2013; Oshkin et al., 2015; Cao et al., 2019). The iron reducers *Geobacter* and *Desulfuromonas* abundance increased as well. Both are well-documented anaerobic iron-reducing bacteria in aquatic sediments and soils (Lovley et al., 2011; An and Picardal, 2015). As expected, the abundance of most methanogens and ANME-1 did not change during the experiment. The decrease in abundance of *Methanothrix* and the order Methanofastidiosales during the experiment suggests that ANME and methanogens are not involved in the observed methanotrophy, as indicated by the geochemical results. The results of the lipid analysis of the 1% O_2_ treatment are in line with the metagenomic results (Figure 5B). There is a similar pattern of the PLFAs to the one previously observed in an anaerobic incubation experiment (Bar-Or et al., 2017) but given the apparent amount of new production in iC_15:0_ (1.3 ng ^13^C/g dw) we can speculate that this is the result of concomitant heterotrophic activity (Aepfler et al., 2019).

### 4.2. The role of methanol in Lake Kinneret sediments

To explain the results of the previous anaerobic incubation experiment (Bar-Or et al., 2017), the production of potential intermediates that can be channeled from archaea to aerobic methanotrophs was suggested. As the most probable candidate is methanol, its role was tested in anoxic methane-generating sediments in two slurry incubation experiments. Our results indicate that under anaerobic conditions, methanol additions fueled methylotrophic methanogenesis rather than methanol oxidation, as opposed to the fatty acids analyses of the 1% O_2_ experiment (Figure 5B; Supplementary Figure S8) and previous observations (Bar-Or et al., 2017). In general, methanol addition increased methane concentrations and higher δ^13^C_DIC_ values, originating from methylotrophic methanogenesis. This aligns with the strong ^13^C-enrichment of methylotrophic methanogen lipids (i.e., phytane and phytenes; Table 2; Supplementary Table S5). These isoprenoid hydrocarbons are indirect indicators of archaeol and hydroxyarchaeols produced by methylotrophic methanogens of the order *Methanosarcinales* (Sprott et al., 1993). Accordingly, the 16S rRNA sequencing results fit the biogeochemical observations and show changes in the relative abundance of various methanogens during the experiment. These ranged from acetoclastic and hydrogenotrophic methanogens, i.e., *Methanosaeta*, *Methanoregula,* and *Methanolinea* (Jetten et al., 1992; Oren, 2014), to methylotrophic methanogens of the genera *Methanosarcina, Methanomethylovorans,* and *Methanomethylicus* (Smith, 1978; Ranalli, 1986; Lomans, 1999; Vanwonterghem, 2016; Supplementary Figures S5A, S6A). In the methanol treatment (experiment D), there was just a slight ^13^C-enrichment and hence an incorporation of methanol into bacterial fatty acids (Table 2). Nonetheless, this suggests that methanol addition also stimulated the activity of bacteria, either directly as a substrate or by consuming ^13^CO_2_ derived from methylotrophic methanogenesis, as expected in this kind of environment (Dijkhuizen and Harder, 1984).

In experiment E, some slurries were amended with BES and hematite, in addition to ^13^C-methanol, to test the potential of methanol turnover when methanogenesis is inhibited or additional electron acceptors available. Similarly, high δ^13^C_DIC_ values in these treatments (Figure 7) suggest the direct oxidation of methanol by bacteria, which is also reflected in higher ^13^C-incorporations compared to those observed during the methanol-only experiment (Table 2). Even though the FA C_16:1ω7_ is associated with methylotrophs (Guckert et al., 1991), they were not detected in the 16S rRNA sequencing results (Supplementary File 1). Alternatively, this FA could indicate the involvement of aerobic methanotrophs; however, their relative abundance decreased compared to the *t_0_* treatment (Supplementary Figure S6). Considering the increase in Fe(II) concentrations, these new production patterns of bacterial fatty acids may indicate the activity of iron reducers (Teece et al., 1999; Zhang et al., 2003). When methanogenesis was inhibited, there was a marginal ^13^C-enrichment in phytane and phytenes compared to the original values in the sediment (−32‰, Vigderovich et al., 2022). These results indicate that methylotrophic methanogens very likely outcompete heterotrophic bacteria for methanol in a natural system.

Hematite addition with and without methanol mostly encouraged net iron reduction. According to the δ^13^C_DIC_ results, hematite does not appear to influence methanol oxidation. However, the isotopic composition of bacterial fatty acids revealed a more complex picture. It seems that the addition of hematite doubled the activity of the heterotrophic bacteria (Table 2), indicating a shortage of electron acceptors in the slurry. The sequencing analysis showed a similar picture, where the relative abundance of several bacteria capable of iron reduction (i.e., *ca. Omnitrophus*, *Anaeromyxobacter,* and *Thermoanaerobaculum*) increased during the incubation time. Similar results were noted for the phylums Zixibacteria and Sva0485. Prior reports identified iron-reducing genes in both members (Treude, 2003; Kerin, 2006; Losey, 2013; Tan, 2019; Garber, 2020; Casar, 2021; Williams, 2021). Interestingly, the type I aerobic methanotroph family *Methylococcaceae* was found in all treatments; however, it only increased in the methanol and hematite treatment. Members of this family have been previously found in suboxic and anoxic environments, such as lake sediments (Bar-Or et al., 2015; Martinez-Cruz et al., 2017; Elul et al., 2021; Su et al., 2022) and in the anoxic hypolimnion of freshwater lakes (Blees et al., 2014; Oswald et al., 2016a; Rissanen et al., 2021). This increase could indicate a potential metabolism that can sustain these methanotrophs in natural anaerobic environments.

## 5. Conclusion

Aerobic methanotrophs were previously discovered in anaerobic methane generating Lake Kinneret sediments. However, their ability to perform the aerobic activity and its potential link to iron reduction as the AOM in these sediments is unclear. By injecting different oxygen concentrations, we show that methanotrophs *Methylomonas* and *Methylobacter* are activated under low levels of oxygen (i.e., 1%). Furthermore, adding oxygen promoted an unexpected increase in net iron reduction in hypoxic slurries. We propose this may occur due to one or a combination of the following processes (i) ferrous iron recycling by its aerobic oxidation to low crystalline minerals available for reduction, (ii) methanotrophs switch from oxygen to iron reduction metabolism when oxygen concentrations are low, and (iii) the methanotrophs’ activity promotes iron reduction by excretion of metabolites, which can be used as Fe(II)-binding ligands. By testing whether methanotrophic bacteria can operate under anoxic conditions we show that methanol is less likely to act as an intermediate between methanogens and methanotrophs, and that methanotrophs do not incorporate methanol. The results of these incubations indicate that methylotrophic methanogens outcompete heterotrophic bacteria as long as the methanogens are not inhibited. Adding hematite to the incubation stimulates iron reducers and possibly methanotrophic bacteria, while methylotrophic methanogens are active. Our findings open new avenues for elucidating microbial networks in reducing environments and indicate that methanotrophs and methylotrophs sustain anaerobic conditions from which they can be revived.

## Data availability statement

The raw reads generated in this study have been deposited in the European Nucleotide Archive (ENA) Database (https://www.ebi.ac.uk/ena/browser/home) as BioProject accession number PRJEB59988. Additional data are available under the Supplementary material and Supplementary Data sections.

## Author contributions

HV, WE, and OS designed the research. HV, ME, MR-B, AG, and OB analyzed the samples and the data. WE and OS supervised HV and provided resources and funding. HV and OS synthesized the data and wrote the original draft. All authors contributed to the article and approved the submitted version.

## Funding

This research work was supported by ERC Consolidator (818450) and Israel Science Foundation (857–2016) grants awarded to OS. Funding for ME was provided by the Deutsche Forschungsgemeinschaft (DFG) under Germany’s Excellence Strategy through the cluster of excellence EXC 2077 “The Ocean Floor – Earth’s Uncharted Interface” (project no. 390741601). Funding for MR-B is funded by the Israel Ministry of Science and Technology Grant 001126 and the Israel Ministry of Energy (Grants 219-17-015 and 221-17-002). HV was supported by a student fellowship from the Israel Water Authority and by a short-term post-doctoral scholarship of the Kreitman School.

## Conflict of interest

The authors declare that the research was conducted in the absence of any commercial or financial relationships that could be construed as a potential conflict of interest.

## Publisher’s note

All claims expressed in this article are solely those of the authors and do not necessarily represent those of their affiliated organizations, or those of the publisher, the editors and the reviewers. Any product that may be evaluated in this article, or claim that may be made by its manufacturer, is not guaranteed or endorsed by the publisher.
