# Supplementary material for: Aerobic methanotrophy increases the net iron reduction in methanogenic lake sediments
Source: Front Microbiol. 2023 Jul 27;14:1206414. doi: 10.3389/fmicb.2023.1206414 (PMC10415106; doi:10.3389/fmicb.2023.1206414)
Supplement: Supplementary file 1 [file Data_Sheet_1.zip › fmicb-14-1206414/Supplementary_material.DOCX]

Supplementary Material

Aerobic methanotrophy increases the net iron reduction in methanogenic lake sediments

Hanni Vigderovich^*^, Werner Eckert, Marcus Elvert, Almog Gafni, Maxim Rubin-Blum, Oded Bergman, and Orit Sivan

* **Correspondence:** Corresponding Author: [hannil@post.bgu.ac.il](mailto:hannil@post.bgu.ac.il)

# Supplementary Data

## Statistical analyses for experiment C

**Two-way repeated measures ANOVA Table (type III tests) experiment C**

* ns = non-significant; significance level; * p ≤ 0.05; ** p ≤ 0.01; *** p ≤ 0.001.

Effect DFn DFd F p p<.05 ges

1 Treatment 1 2 54.808 1.80e-02 * 0.917

2 time_series 3 6 134.555 6.83e-06 * 0.897

3 Treatment:time_series 3 6 73.738 3.99e-05 * 0.812

**post hoc Time (t tests, bonferroni correction)**

time Effect DFn DFd F p `p<.05` ges p.adj

1 1 Treatment 1 2 0.15 0.736 "" 0.046 1

2 2 Treatment 1 2 24.2 0.039 "*" 0.864 0.156

3 3 Treatment 1 2 92.6 0.011 "*" 0.969 0.044

4 4 Treatment 1 2 80.7 0.012 "*" 0.941 0.048

**post hoc Treatment (t tests, bonferroni correction)**

Time .y. group1 group2 n1 n2 statistic df p p.adj p.adj.signif

1 0 FeLog O2+Ch4 O2+N2 5 3 1.29 5.97 0.245 0.98 ns

2 11 FeLog O2+Ch4 O2+N2 5 3 7.17 6.00 0.000373 0.00149 **

3 23 FeLog O2+Ch4 O2+N2 5 3 13.0 5.46 0.0000254 0.000102 ***

4 37 FeLog O2+Ch4 O2+N2 5 3 9.00 3.57 0.00142 0.00568 **

**One-way repeated measures ANOVA Table (type III tests) – with Methane**

Effect DFn DFd F p p<.05 ges

1 time 3 12 15.486 0.000199 * 0.315

**post hoc Time (t tests, bonferroni correction)**

.y. group1 group2 n1 n2 statistic df p p.adj p.adj.signif

1 FeLog 0 11 5 5 2.49 5.31 0.052 0.314 ns

2 FeLog 0 23 5 5 2.72 5.75 0.036 0.216 ns

3 FeLog 0 37 5 5 1.92 6.01 0.103 0.618 ns

4 FeLog 11 23 5 5 -0.0829 7.81 0.936 1 ns

5 FeLog 11 37 5 5 -0.770 7.60 0.465 1 ns

6 FeLog 23 37 5 5 -0.749 7.95 0.475 1 ns

**One-way repeated measures ANOVA Table (type III tests) – without Methane**

Effect DFn DFd F p p<.05 ges

1 time 3 6 122.103 9.1e-06 * 0.968

**post hoc Time (t tests, bonferroni correction)**

.y. group1 group2 n1 n2 statistic df p p.adj p.adj.signif

1 FeLog 0 11 3 3 14.1 2.78 0.001 0.007 **

2 FeLog 0 23 3 3 19.7 2.56 0.000719 0.004 **

3 FeLog 0 37 3 3 11.1 2.24 0.005 0.032 *

4 FeLog 11 23 3 3 6.16 3.89 0.004 0.023 *

5 FeLog 11 37 3 3 2.61 3.09 0.077 0.463 ns

6 FeLog 23 37 3 3 -1.87 3.42 0.147 0.882 ns

# Supplementary Figures and Tables

## Supplementary Figures


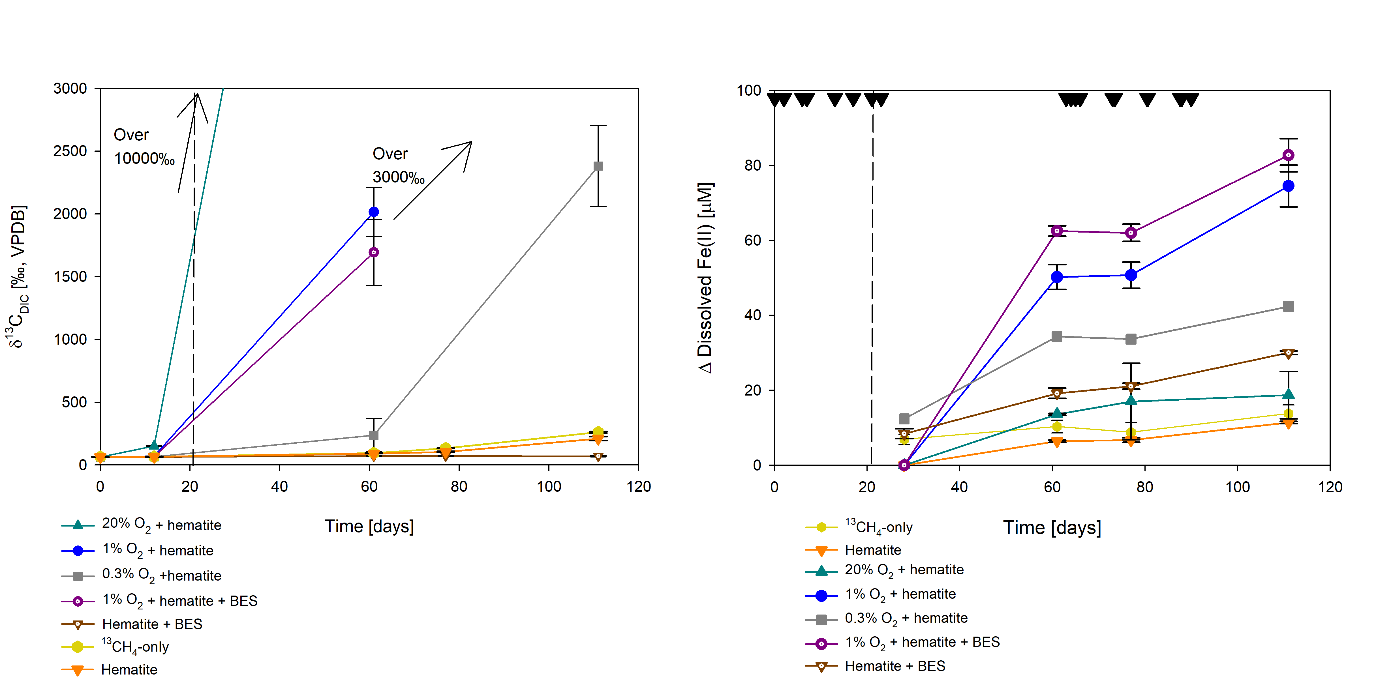


**Supplementary Figure 1**: Development of δ^13^C_DIC_ (A) and ΔFe(II) (B) over time in experiment B, where the participation of methanogens in the methanotrophy was tested by the addition of BES. All non-control bottles were amended with 10 mM hematite, injection of 1 ml ^13^C-labeled methane (after 21 days, at the dashed line), and oxygen injections. Black triangles represent oxygen injection times to the incubations. Error bars represent the average deviation from the mean of duplicate bottles.


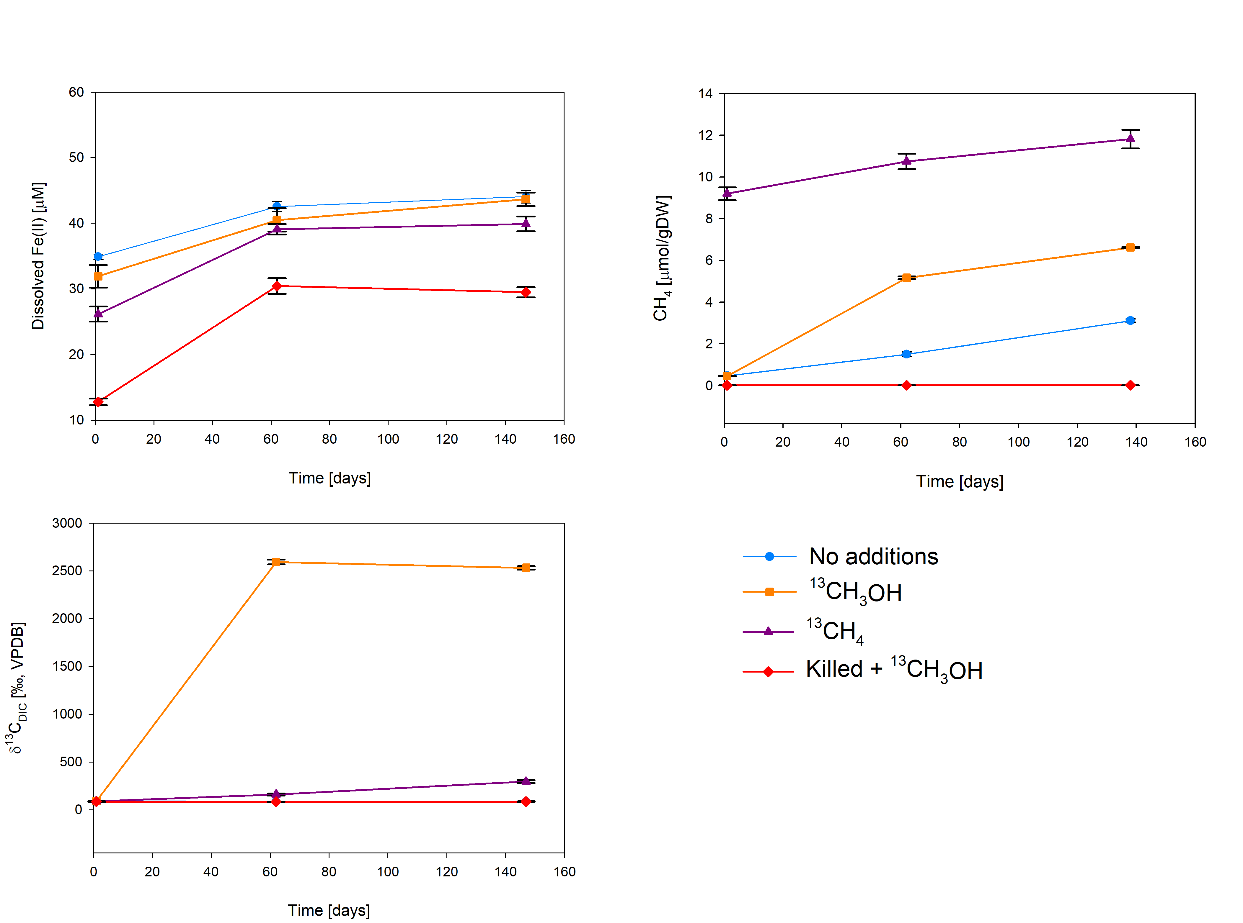


**Supplementary Figure 2**: Development of ΔFe(II) (A), CH_4_ (B), and δ^13^C_DIC_ (C) over time in experiment D with the addition of ^13^C-labeled methanol to test its participation as a substrate for methanogens and methanotrophs. Oxygen was not injected during this experiment. Error bars represent the average deviation from the mean of triplicate bottles.


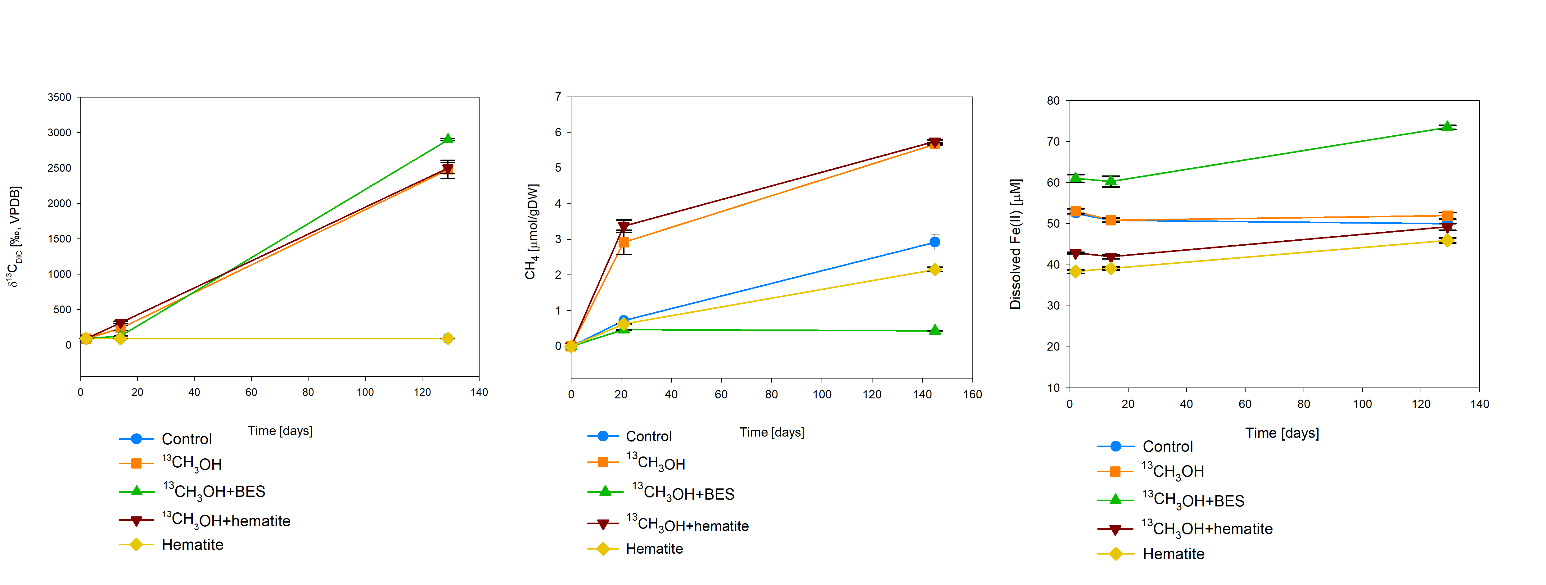


**Supplementary Figure 3**: Development of δ^13^C_DIC_ (A) and ΔFe(II) (B) over time in experiment E with the addition of ^13^C-labeled methanol and BES to test methanol participation as a substrate for the methanotrophs. All non-control bottles were also amended with 10 mM hematite and oxygen injections. Error bars represent the average deviation from the mean of the duplicate bottles.


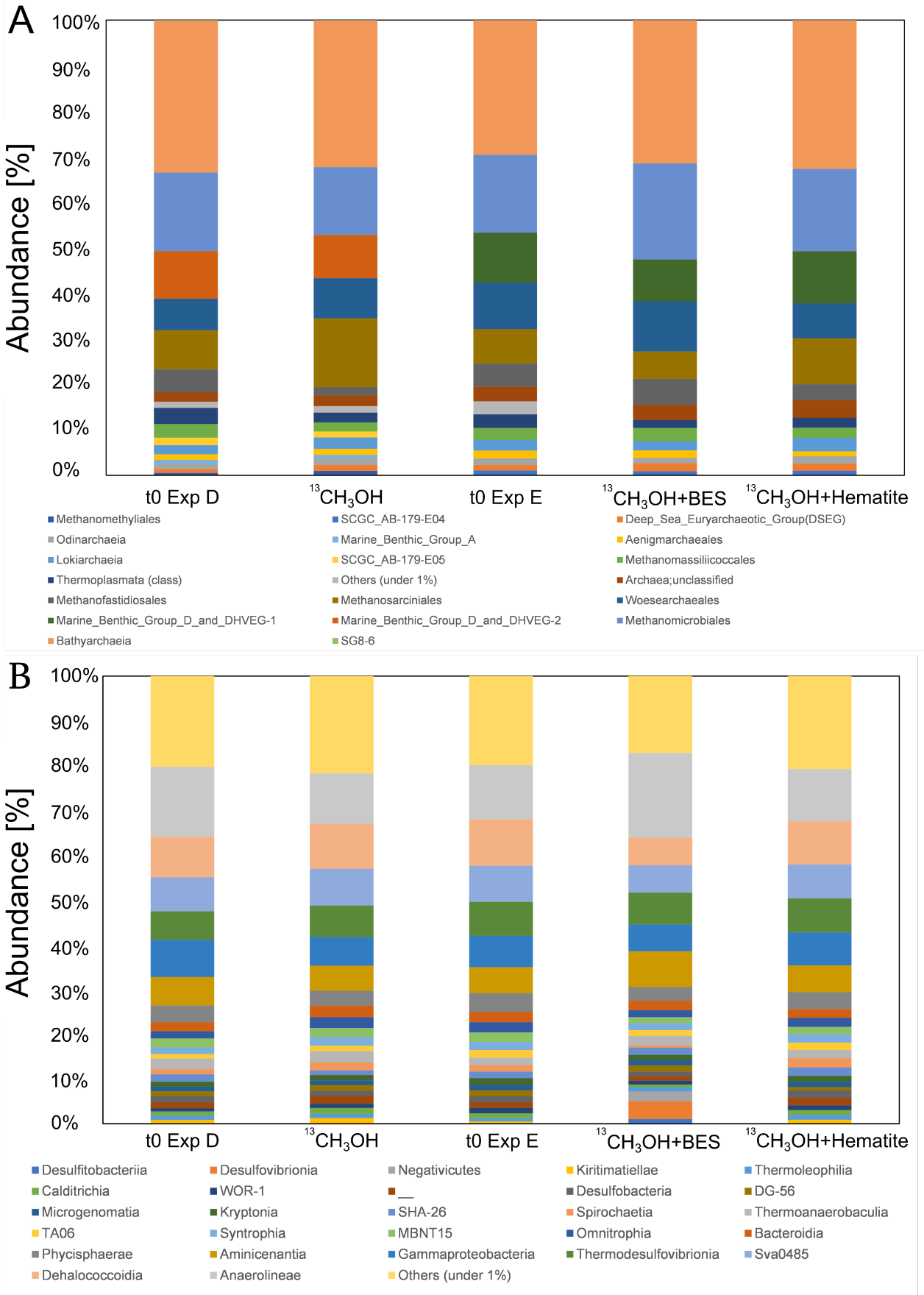


**Supplementary Figure 4:** Microbial community of anoxic slurry incubation experiments D following the addition of ^13^CH_3_OH, and experiment E following the addition of ^13^CH_3_OH+BES and ^13^CH_3_OH+hematite. Relative abundance of archaea at the order level (A) and bacteria at the class level (B) are presented.


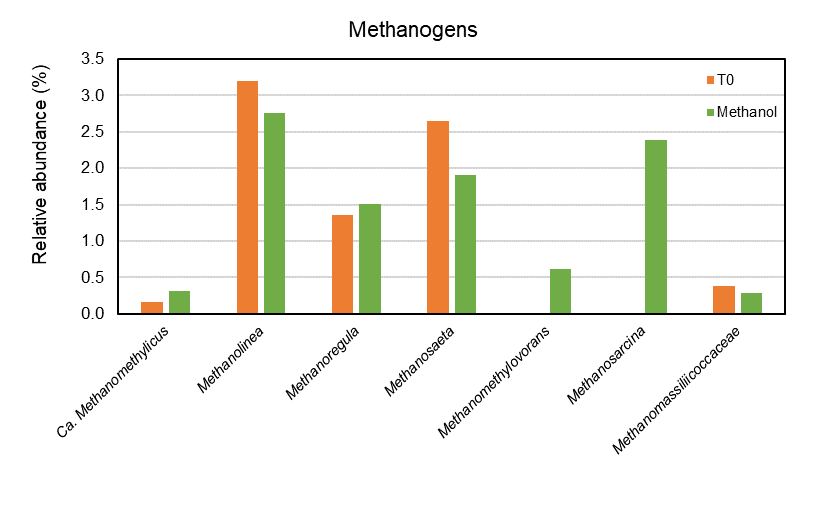


A


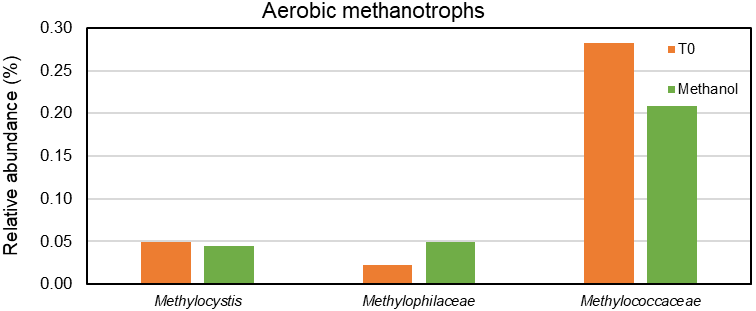


B


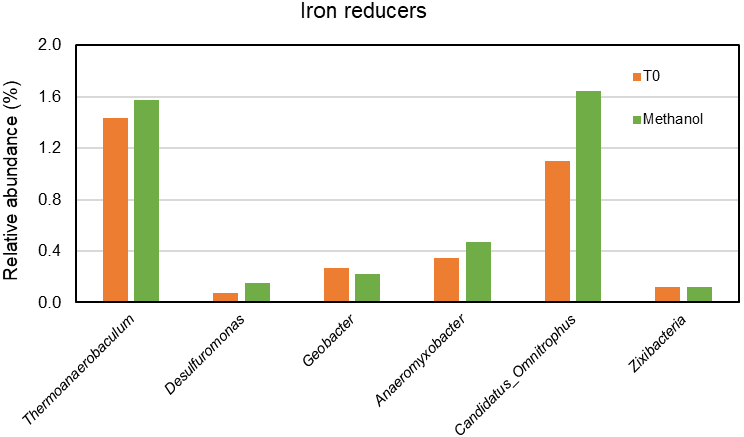


C

**Supplementary Figure 5:** Relative abundance of methanogens (A), aerobic methanotrophs (B), and iron reducers (C) in slurry incubation experiment D where anaerobic conditions were kept (no injection of oxygen), following the addition of ^13^C-labeled methanol. Samples for microbial analysis were taken from experiment D around the same time of the lipid sampling (459 days of incubation).

A

B

C

**Supplementary Figure 6:** Relative abundance of methanogens (A), aerobic methanotrophs (B), and iron reducers (C) in slurry incubation experiment E, following the addition of ^13^C-labeled methanol, BES, and hematite. Samples for microbial analysis around the same time as the lipid sampling (after 284 days of incubation).





**Supplementary Figure 7**: Comparison of newly produced fatty acid patterns (in ng ^13^C/g dw) during experiments D (^13^CH_4_ and ^13^CH_3_OH; after 462 days) and E (^13^CH_3_OH + BES and ^13^CH_3_OH + hematite; after 287 days).

## Supplementary Tables

| Days | Hamatite + O_2_ 1% | Hamatite + O_2_ 0.1% | Hematite | Killed + Hamatite + O_2_ 1% |
| --- | --- | --- | --- | --- |
| 0 | 44.9 | 46.4 | 43.7 | 44.6 |
| 5 | 132.5 | 53.7 | 46.9 | 42.1 |
| 20 | 1044 | 68.2 |  | 25.9 |
| 30 | 1218.4 | 69.5 | 49.5 | 26 |
| 36 | 1536.2 | 70.2 |  | 22.4 |
| 43 | 1746.5 | 72 |  | 18.9 |
| 52 | 2096.1 | 74.2 |  | 15.5 |
| 70 | 2182.7 | 74.9 | 59.5 | 14.3 |

**Supplementary Table 1**: The average values of δ^13^C-DIC [‰, VPDB] during experiment A

**Supplementary Table 2**: The average values of the change in dissolved Fe(II) concentrations [µM] during experiment A

| Days | Hamatite + O_2_ 1% | Hamatite + O_2_ 0.1% | Hematite | **Killed + hamatite + 1%O_2_ |
| --- | --- | --- | --- | --- |
| 0 | 0 | 0 | 0 | 0 |
| 5 | 0.8381 | 4.3656 | 0.3215 | 4.3194 |
| 30 | 30.4023 | 4.824 | 10.1264 | 17.9347 |
| 44 | 37.3454 | 6.6956 |  | 14.2995 |
| 70 | 51.0065 | 11.1218 | 16.6634 | 23.8668 |

**Supplementary Table 3**: Values of δ^13^C-DIC [‰, VPDB] during experiment B

| Days | ^13^CH_4_ | 0% O2 + hematite | 0% O_2_ + hematite + BES | 0.3% O_2_ + hematite | 1% O_2_ + hematite | 1% O_2_ + hematite + BES | 20% O_2_ + hematite | Killed + 1% O_2_ + hematite + BES |
| --- | --- | --- | --- | --- | --- | --- | --- | --- |
| 0 | 65.9 | 63.6 | 64.3 | 63.4 | 63.7 | 63.8 | 63.8 | 58 |
| 12 | 66 | 65.1 | 64.1 | 65 | 69.4 | 66.9 | 150.8 | 52 |
| 61 | 95.6 | 90.1 | 72.7 | 237.1 | 2015.7 | 1692.8 | 9225.8 | 46.5 |
| 77 | 134.4 | 105 | 72.1 |  |  |  |  | 48.3 |
| 111 | 260.9 | 210.2 | 70.8 | 2381 |  |  |  | 31.2 |

| Days | ^13^CH_4_ | 0% O_2_ + hematite | 0% O_2_ + hematite + BES | 0.3% O_2_ + hematite | 1% O_2_ + hematite | 1% O_2_ + hematite + BES | 20% O_2_ + hematite | **killed+1% O_2_ + hematite + BES |
| --- | --- | --- | --- | --- | --- | --- | --- | --- |
| 28 | 6.9 | 0 | 8.4 | 12.3551 | 0 | 0 | 0 | 1.8 |
| 61 | 10.3 | 6.4 | 19.2 | 34.2996 | 50.2 | 62.5 | 13.6 | 24.4 |
| 77 | 8.8 | 6.7 | 21.1 | 33.629 | 50.7 | 62 | 17 | 20.7 |
| 111 | 13.8 | 11.4 | 30 | 42.3878 | 74.5 | 82.7 | 18.7 | 28.8 |

**Supplementary Table 4**: The average values of the change in dissolved Fe(II) concentrations [µM] during experiment A

**It appears that by autoclaving abiotic iron reduction of siderite was activated, which resulted in an increase of dissolved Fe(II) and a decrease of the δ^13^C-DIC values. This was observed in both A and B experiments.

**Supplementary Table 5**: Complete isotopic pattern (in ‰) of diagnostic bacterial fatty acids and archaeal-derived phytane, phytene, and biphytane compared to DIC as an overall indicator of methane/methanol turnover. n.d.: not detected.

| Treatment | i14:0 | 14:0 | i15:0 | ai15:0 | 15:0 | i16:0 | 16:1ω7 | 16:1ω5 | 16:0 | 10Me16:0 | i17:0 | ai17:0 | 17:0 | 18:1ω9 | 18:1ω7 | 18:0 | Phytane | Phytene | Biphytane |
| --- | --- | --- | --- | --- | --- | --- | --- | --- | --- | --- | --- | --- | --- | --- | --- | --- | --- | --- | --- |
| ^13^CH_4_ | -22 | -27 | -27 | -21 | -33 | -19 | -42 | -42 | -29 | -34 | -29 | -24 | -32 | -27 | -26 | -30 | -3 | -22 | -32 |
| ^13^CH_3_OH | 21 | -16 | 170 | 130 | -28 | 50 | -7 | -29 | -22 | -10 | 35 | 88 | -17 | -27 | 4 | -23 | 2300 | 530 | 6 |
| ^13^CH_3_OH+BES | 170 | 61 | 710 | 410 | 81 | 220 | 850 | 310 | 31 | 230 | 500 | 260 | 94 | 39 | 340 | 36 | -5 | -31 | -14 |
| ^13^CH_3_OH+Hematite | 55 | -3 | 710 | 340 | -32 | 100 | 140 | 36 | -10 | -20 | 128 | 170 | -9 | -8 | 130 | -19 | 1600 | 360 | 27 |
| ^13^CH_4_+1%O_2_ | 88 | 660 | 480 | 280 | 140 | 160 | 4100 | 4200 | 7800 | 130 | 110 | 58 | 170 | -29 | 14 | 220 | -4 | n.d. | 3 |
